# Supplementary material for: De novo variants in LDB1 are linked to distinct neurodevelopmental phenotypes determined by variant location and differing pathomechanisms
Source: Am J Hum Genet. 2026 Jun 19;113(7):1543–57. doi: 10.1016/j.ajhg.2026.05.012 (PMC13504347; doi:10.1016/j.ajhg.2026.05.012)
Supplement: Supplementary file 1 [file mmc1.pdf]

The American Journal of Human Genetics, Volume *113*

Supplemental information

De novo variants in *LDB1* are linked to distinct neurodevelopmental phenotypes determined by variant location and differing pathomechanisms

Rebecca Fluri, Mireia Coll-Tané, Theresa Brunet, Benjamin Cogne, Solene Conrad, Mathilde Nizon, Francesco Nicita, Lorena Travaglini, Antonio Novelli, Margie Glissmeyer, Amanda Peterson, Jillian G. Buchan, Dan Serber, Kolja Meier, Jutta Gärtner, Susann Diegmann, Veronique Pingault, Tania Attie-Bitach, Thomas Courtin, Michael C. Schneider, Wing Hung, Inderneel Sahai, Lauren O'Grady, Katharina Steindl, Sarju G. Mehta, Christel Depienne, Delphine Heron, Boris Keren, Solveig Heide, Shane McKee, Franco Laccone, Lisa M. Dyer, Catherine Melver, Connie Motter, Wendy D. Jones, Zoey Trueblood Wilson, Divya Vats, Kristina Huß, Christiane Zweier, Heinrich Sticht, and Anne Gregor

Supplemental Information

Supplemental Figures

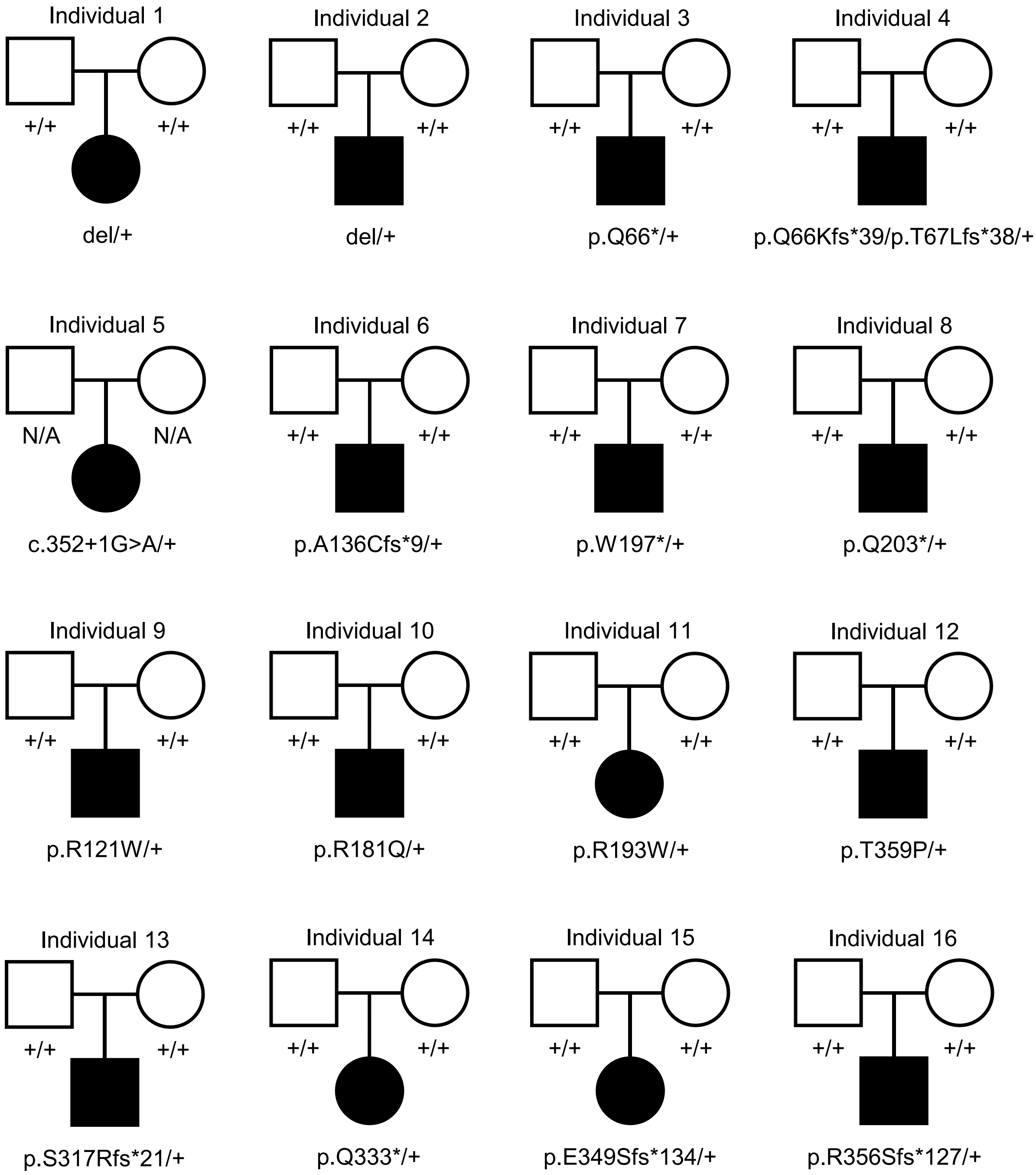

Figure S1. Pedigrees of all affected individuals included in this study.

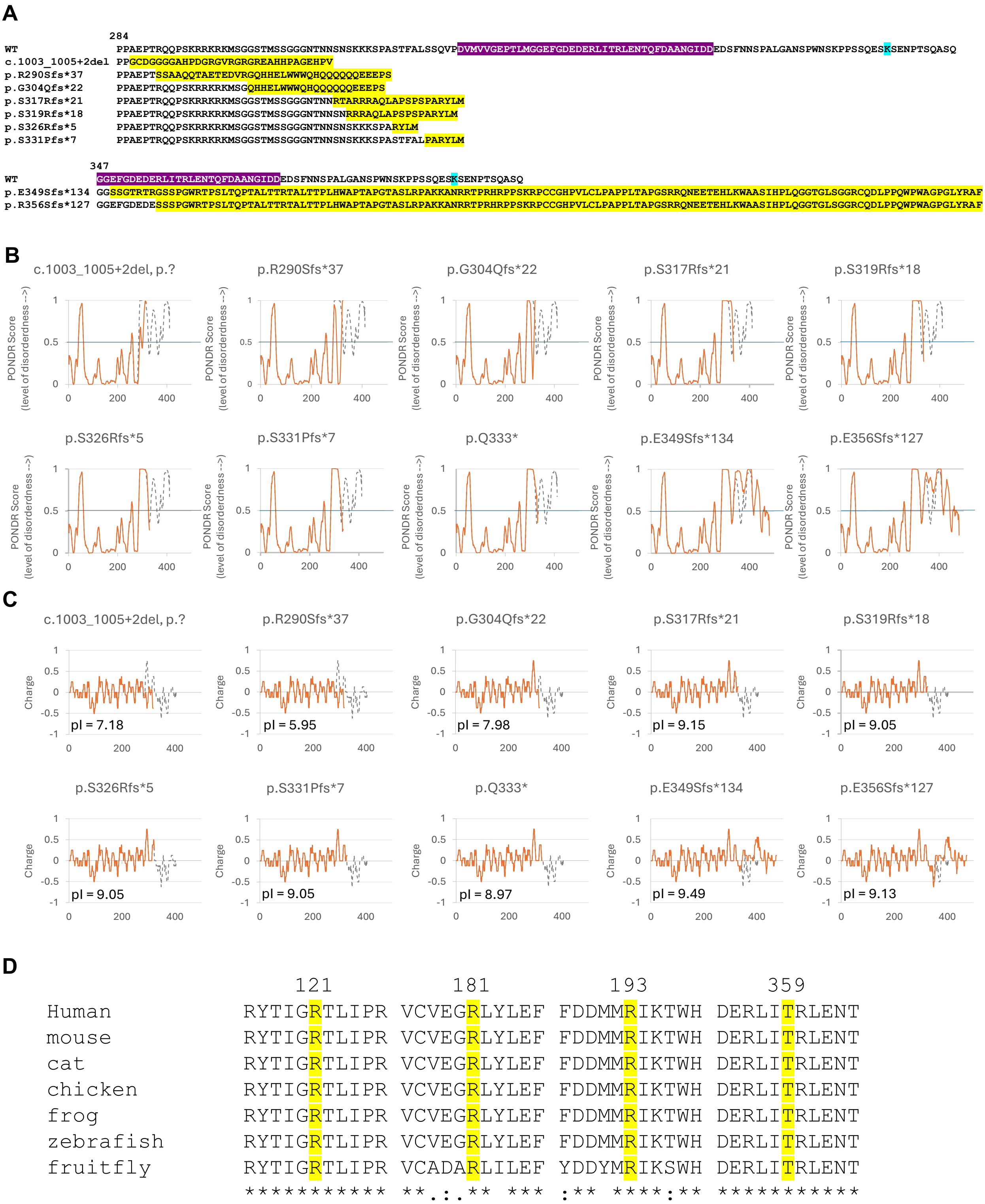

Figure S2. Changed C-terminal tail upon C-terminal LID-disrupting variants and conservation of *LDB1* missense variant positions.

(A) Published and novel C-terminal LID-disrupting frameshift variants alter C-terminal tails of LDB1 (alterations highlighted in yellow for each variant), omitting the ubiquitination site (marked in blue in the wild-type (WT) sequence) and at least part of the LID (highlighted in purple).

(B) Many C-terminal LID-disrupting variants reduce disorderdness of the C-terminus. Analysis was done using PONDR. Wild-type is shown as grey dashed line, respective mutants in orange.

(C) Charge analysis shows that most C-terminal LID-disrupting variants reduce acidity of LDB1 and increase the isoelectric point of the protein ((pI); pI wild-type LDB1 = 6.52). Analysis was done using the EMBOSS Charge tool for protein charge and the Expasy compute pI tool for isoelectric point. Wild-type is shown as grey dashed line, respective mutants in orange.

(D) All identified missense variants in LDB1 affect residues that are highly conserved. Multiple species alignment was done with Clustalw.[1] Reference sequences used were: human: NP_001106878.1, mouse: NP_001106879.1, cat: XP_023096621.1, chicken: XP_046775883.1, frog: NP_001090450.1, fruit fly: AAB62574.1. Affected amino acids are highlighted in yellow.

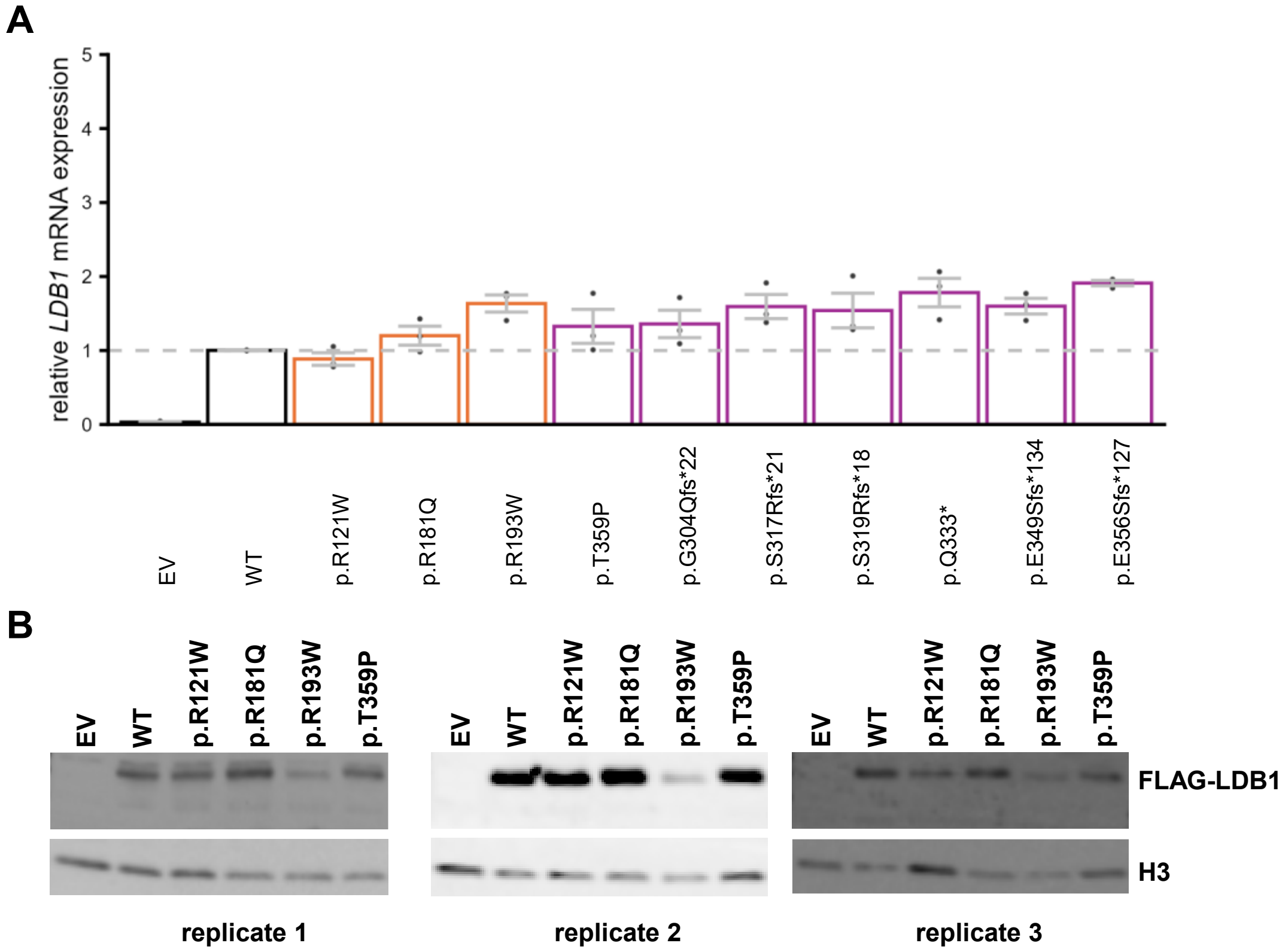

Figure S3. Expression level analysis of *LDB1* variants.

(A) Transcript levels of transfected wild-type (WT) or mutant *LDB1* were mostly comparable between different constructs and did not correlate with protein levels (see Figure 3B). QPCR analysis was carried out in three biological replicates with four technical replicates each; data were normalized to expression of WT *LDB1*; individual data points (biological replicates) are shown as black dots. EV – empty vector.

(B) Blots of three biological replicates of protein analysis for WT LDB1 and missense variants without MG132 treatment show consistent reduction of LDB1 for missense variant p.(Arg193Trp). Quantification and a representative image with the fourth replicate are shown in Figure 3B.

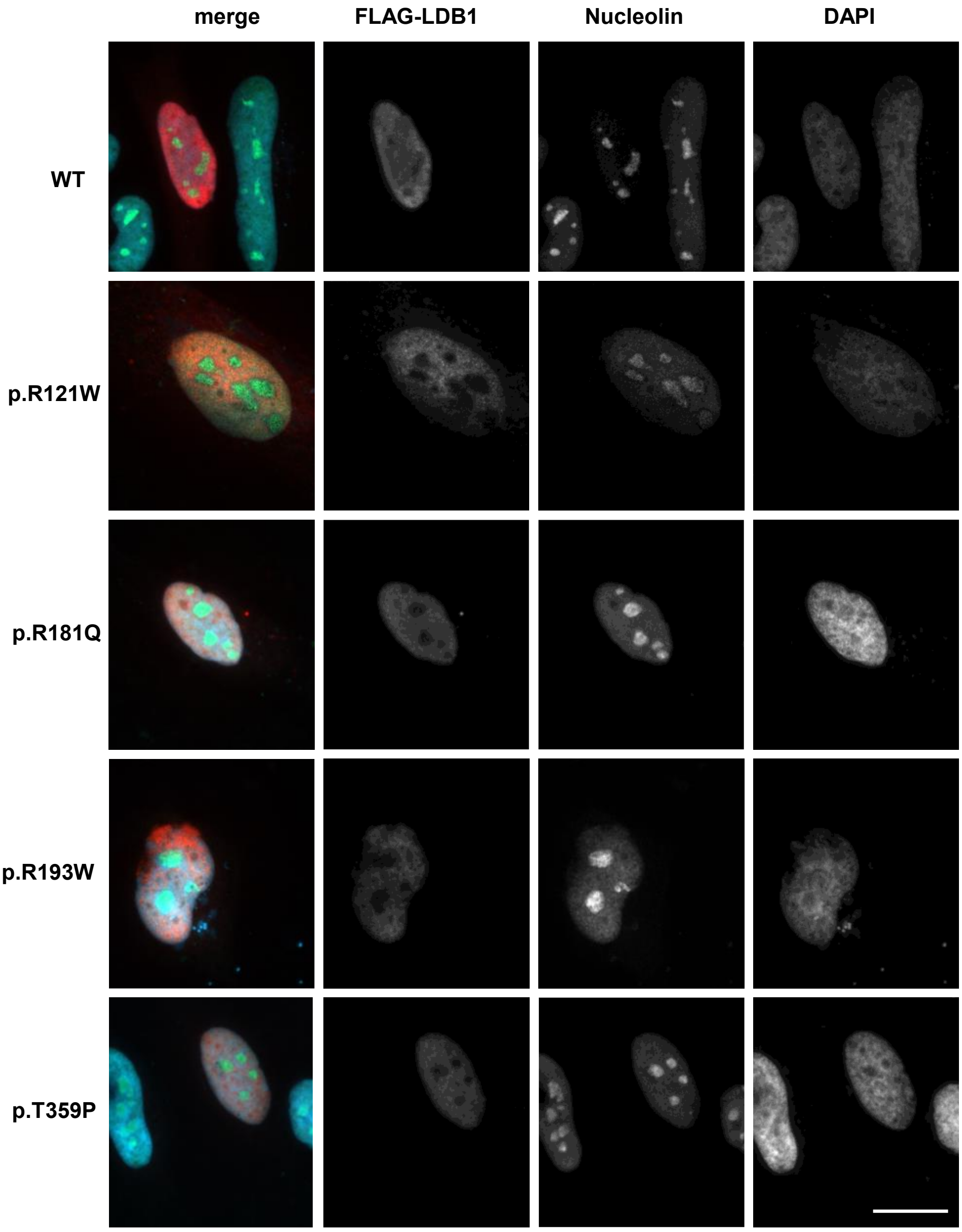

Figure S4. Immunofluorescence analysis of LDB1 missense variants.

Diffuse cytoplasmic localization of overexpressed FLAG-LDB1 in the nucleus of HeLa cells is not affected by LDB1 missense variants. Cells were stained with anti-FLAG (red) and anti-nucleolin (green) antibodies and nuclei were counterstained with DAPI. At least 50 cells were analyzed per condition. Images were taken on a Axioimager Z2 with a 63x objective, scale bar = 20µm.

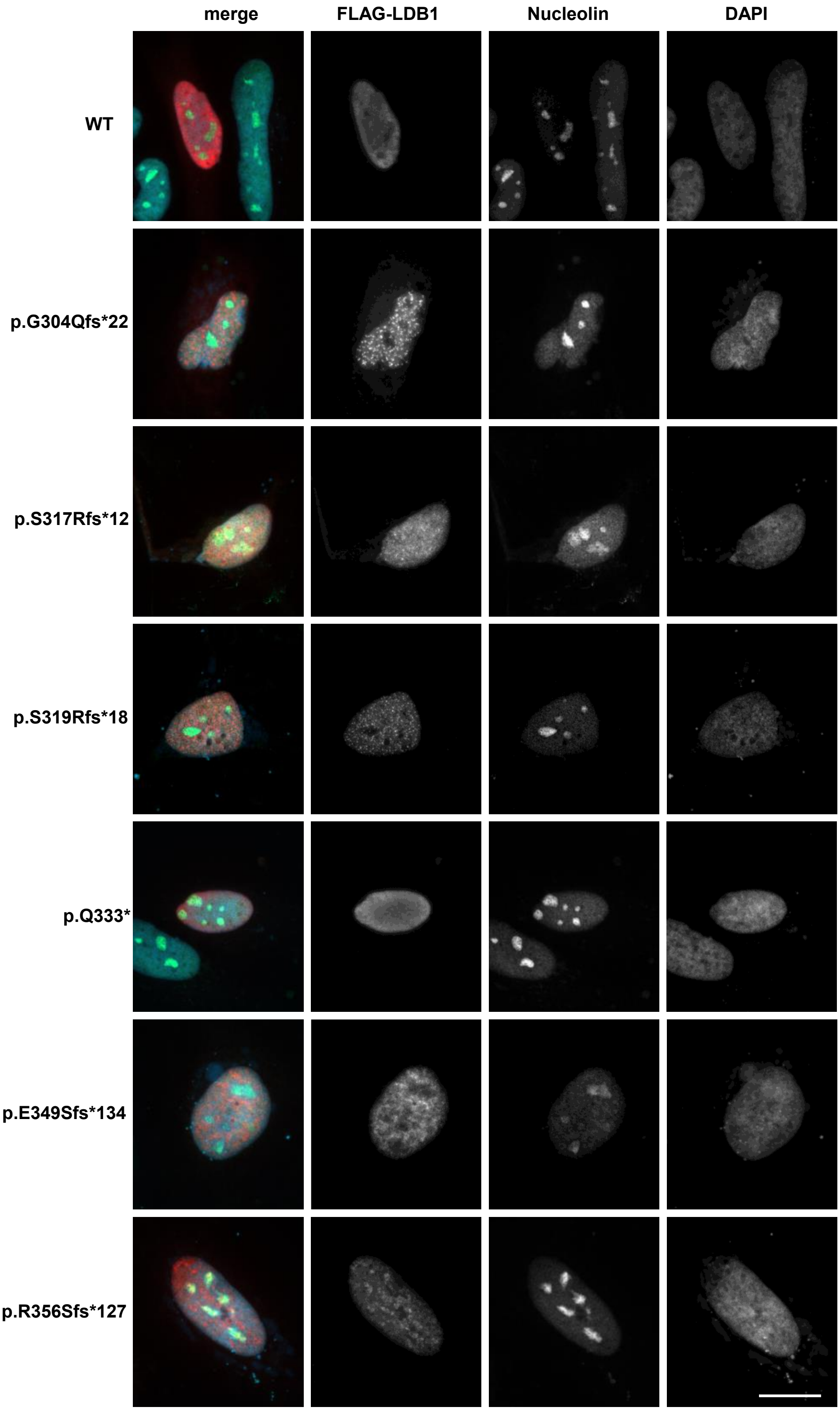

Figure S5. Immunofluorescence analysis of C-terminal LID-disrupting LDB1 variants.

All C-terminal frameshift variants show punctate nuclear aggregates of LDB1 that do not co-localize with the nucleolus. No aggregates are found for the nonsense variant p.(Gln333*). Cells were stained with anti-FLAG (red) and anti-nucleolin (green) antibodies and nuclei were counterstained with DAPI. At least 50 cells were analyzed per condition. Images were taken on a Axioimager Z2 with a 63x objective, scale bar = 20µm.

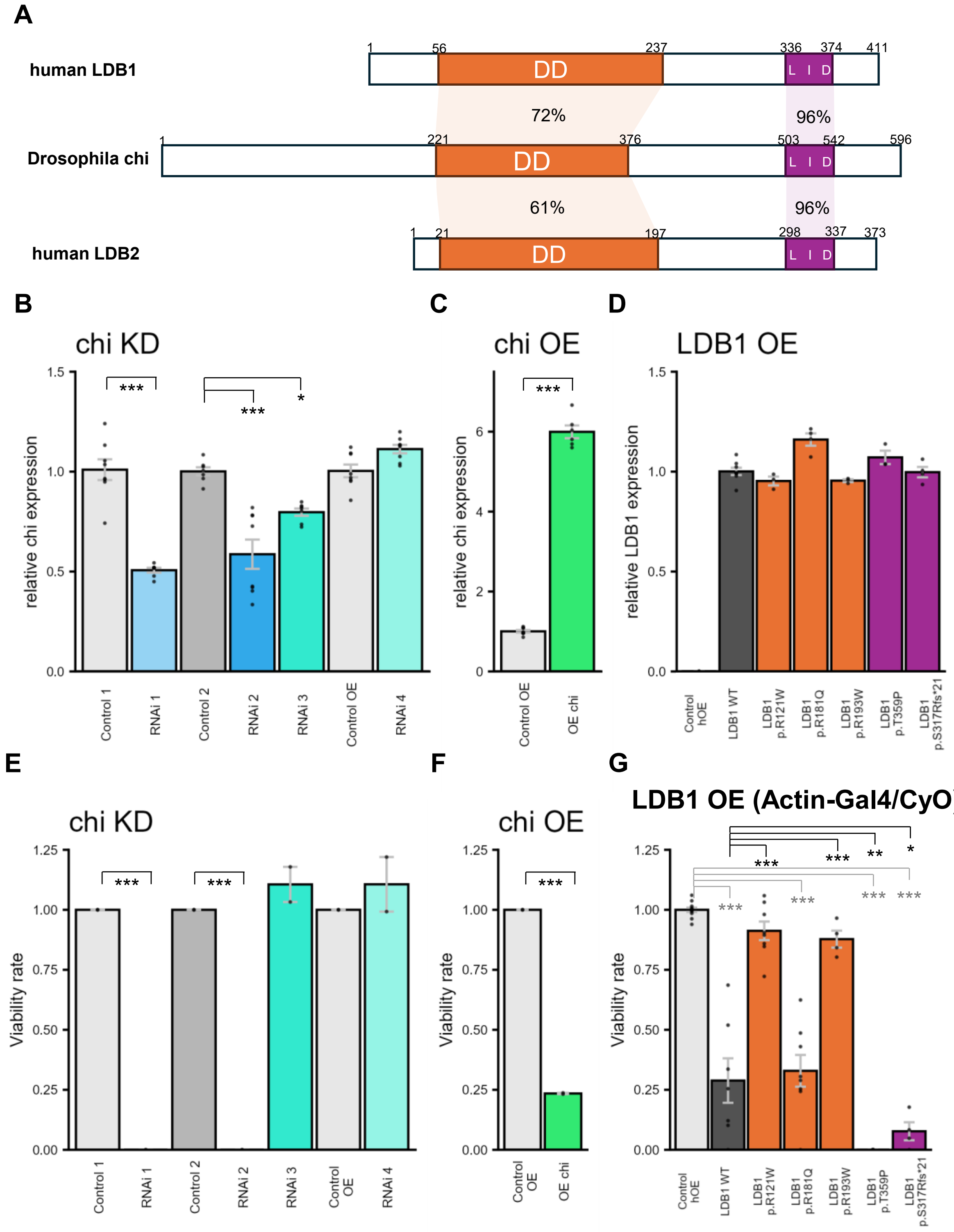

Figure S6. Expression and viability analysis of *chi/LDB1 Drosophila* lines.

(A) Schematic overview of human LDB1 and LDB2 as well as *Drosophila* chi, showing conservation of functional domains. DD – dimerization domain, LID – LIM interacting domain.

(B-D) Expression levels of *chi/LDB1* upon ubiquitous dosage manipulation were measured using qPCR. Knockdown of *chi* was confirmed for RNAi 1 and 2 (B) and overexpression of *chi* was confirmed for OE chi (C). Overexpression levels of human wildtype and mutant *LDB1* are

comparable for all lines (D). Analysis was carried out in at least 4 technical replicates; data were normalized to respective references: RNAi 1 to Control 1, RNAi 2 & 3 to Control 2, RNAi 4 & OE chi to Control OE and for overexpression of human LDB1 to LDB1 WT (see Table S3 for description of lines and matching controls); individual data points are shown as black dots and significance in B) & C) was calculated using a 1-sample student's t-test with a theoretical value of 1 (* $p < 0.05$, *** $p < 0.001$).

(E-F) Viability analysis of ubiquitous *chi* knockdown (E) or overexpression (F) with the actin-GAL4 / Tm3 Sb Tb driver showed complete lethality for RNAi lines 1 and 2 (E) and partial lethality for OE chi (F). Viability was not affected in RNAi 3 and 4, consistent with less or absent knockdown of *chi* (see (B)). Viability was normalized to that of respective Control lines set to 1. Significance was calculated using a student's t-test on the ratio of knockdown/overexpression vs. Balancer flies (*** $p < 0.001$).

(G) Confirmation of lethality phenotype of overexpression of human LDB1 with a second driver line in addition to data shown in Figure 4C. Viability of male flies upon ubiquitous overexpression (using actin-GAL4 /CyO driver) of human wild-type of mutant LDB1 was reduced for wild-type and p.(Arg181Gln), and even more strongly reduced for variants p.(Thr359Pro) and p.(Ser317Argfs*21). Viability was normalized to the Control line (Control hOE), which was set to 1. The experiment was carried out independently at least three times in duplicates each. At least 100 flies were counted per experiment and genotype. Significance was calculated on the ratios of non-CyO (overexpression) vs. CyO (Balancer) flies using a student's t-test with Bonferroni correction for multiple testing (* $p < 0.05$, ** $p < 0.01$, *** $p < 0.001$).

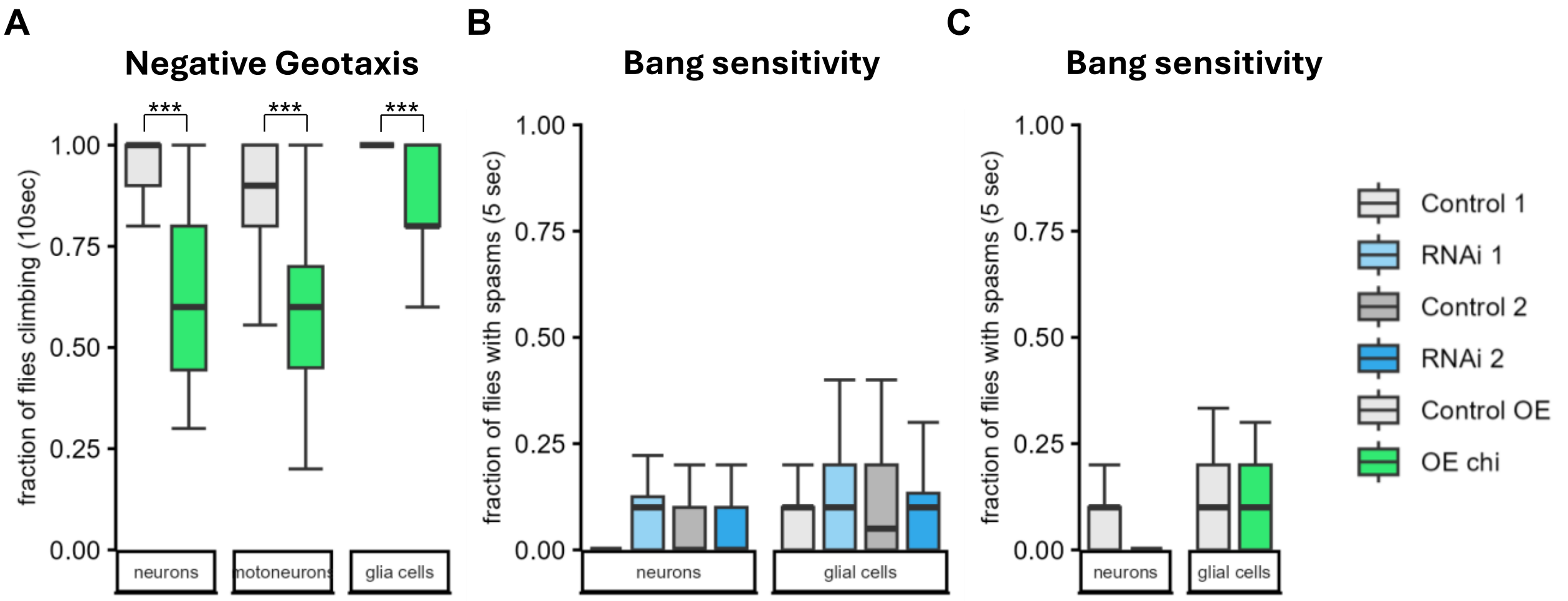

Figure S7. Negative geotaxis and bang sensitivity results of upon *chi* knockdown and overexpression.

(A) Climbing ability as measured with negative geotaxis assay was impaired upon pan-neuronal, motoneuron-specific and glial overexpression of *chi*. Fraction of flies able to climb 8 cm in 10sec after being tapped to the bottom of the vial was assessed.

(B-C) Seizure susceptibility is not increased upon pan-neuronal or glial knockown (B) or overexpression (C) of *chi* as measured with bang sensitivity assay. Fraction of flies showing spasms or paralysis 5 sec after 10sec of vortexing was measured.

At least 100 flies in batches of 10 (n= 10) were tested per experiment and condition. Significance was calculated using a Wilcoxon rank-sum test (*** $p < 0.001$).

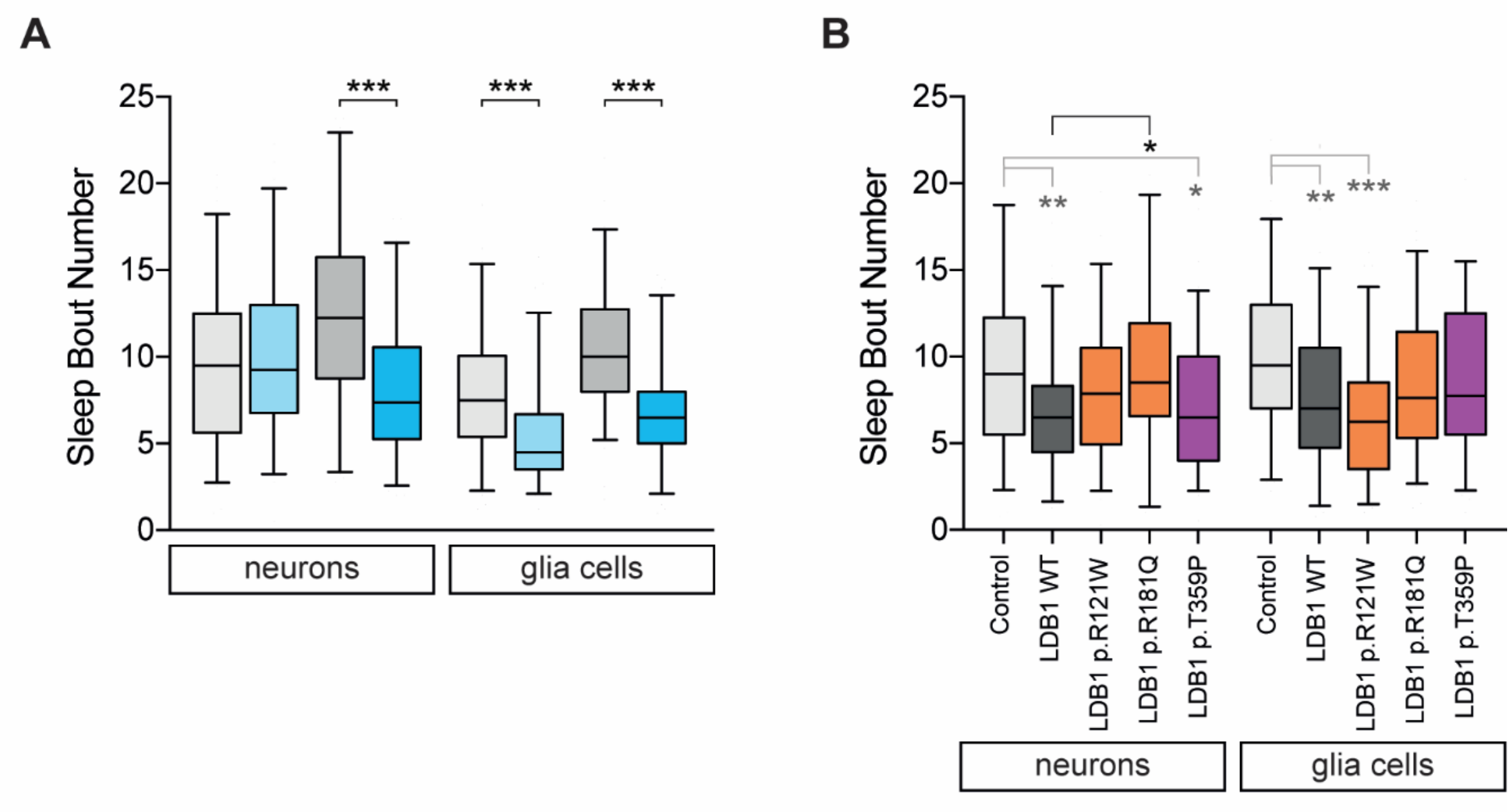

Figure S8. Sleep analysis shows alterations of sleep pattern upon *chi* knockdown and *LDB1* overexpression.

(A) Average number of sleep bouts during the dark period (ZT12-24). Pan-neuronal *chi* knockdown males with RNAi 2 exhibit fewer sleep bouts compared to isogenic controls. RNAi 1 shows no significant difference relative to controls. *Chi* knockdown in glial cells using both RNAi lines results in fewer sleep episodes compared to their respective background controls.

(B) Average number of sleep bouts during the dark period (ZT12–24). Neuronal overexpression of wild-type *LDB1* reduces sleep bouts compared to controls. Pan-neuronal overexpression of LDB1 carrying the R181Q variant significantly increases sleep bout frequency compared to wildtype LDB1 overexpression. Glial overexpression of wild-type LDB1 and the R121W variant reduces sleep bouts compared to controls. Data are represented as boxplots (25th-75th percentiles, median line; whiskers indicate 5th-95th percentiles). At least n = 40-70 flies were tested per genotype. Mann-Whitney or ANOVA tests, with Bonferroni correction for multiple comparisons. Significance: * $p < 0.05$, ** $p < 0.01$, *** $p < 0.001$.

Supplemental Tables

Table S1. Overview of detailed molecular and clinical information on affected individuals.

See excel file Table S1_S4.

Table S2. Overview of all antibodies used in this study

Target	Species	Catalog #	Supplier	Dilution
FLAG	rabbit	F7425	Sigma-Alrich	1:5’000 (WB)
FLAG	mouse	F1804	Sigma-Aldrich	1:500 (IF)
Myc	rabbit	2272	CST	1:2’500 (WB)
HA	rabbit	3724	CST	1:2’000 (WB)
Histone H3	rabbit	4499	CST	1:10’000 (WB)
Nucleolin	rabbit	14574	CST	1:100 (IF)
anti-rabbit-HRP	goat	170-6515	Bio-Rad	1:15’000
Alexa Fluor 488 anti-rabbit	goat	A11008	Thermo	1:500 (IF)
Alexa Fluor 546 anti-mouse	donkey	A10036	Thermo	1:500 (IF)

Table S3. Overview of all *Drosophila* lines used in this study

Fly Line	Description	Order #	Vendor
RNAi 1	knockdown of chi	107314	VDRC
RNAi 2	knockdown of chi	31049	BDSC
RNAi 3	knockdown of chi	35435	BDSC
RNAi 4	knockdown of chi	30454	VDRC
OE chi	overexpression of chi	67741	BDSC
Control 1	control for RNAi 1	60100	VDRC
Control 2	control for RNAi 2 and 3	36303	BDSC
Control OE	control for OE chi and RNAi 4	60000	VDRC
Control hOE	control for human OE lines	24749	BDSC
LDB1 WT	human OE	custom-made	FlyORF
LDB1 p.R121W	human OE	custom-made	FlyORF
LDB1 p.R181Q	human OE	custom-made	FlyORF
LDB1 p.R193W	human OE	custom-made	FlyORF
LDB1 p.T359P	human OE	custom-made	FlyORF
LDB1 p.S317Rfs*21	human OE	custom-made	FlyORF
RNAi 1 ; LDB1 WT	human OE and chi KD	custom-made	assembled in-house
RNAi 1 ; LDB1 p.R121W	human OE and chi KD	custom-made	assembled in-house
RNAi 1 ; LDB1 p.R181Q	human OE and chi KD	custom-made	assembled in-house
RNAi 1 ; LDB1 p.T359P	human OE and chi KD	custom-made	assembled in-house
Actin-GAL4/Tm3 Sb Tb	ubiquitous driver	3954	BDSC
Actin-GAL4/CyO	ubiquitous driver	4414	BDSC
Elav-GAL4/CyO	pan-neuronal driver, used for Geotaxis and Bang sensitivity	8765	BDSC
Elav-GAL4	pan-neuronal driver, used for sleep analysis	8760	BDSC
D42-GAL4	motoneuron driver	8816	BDSC
Repo-GAL4/Tm3 Sb	glial driver, used for Geotaxis and Bang sensitivity	7415	BDSC
Repo-GAL4/Tm6 Sb Tb	glial driver, used for sleep analysis	re-balanced from 7415	assembled in-house
Kr/Cyo ; D/Tm6c Sb Tb	double balancer line for generation of rescue constructs		assembled in-house

KD – knockdown, OE – overexpression, hOE – human gene overexpression, WT – wild-type

Table S4. Overview of detailed molecular data for all variants including applied ACMG criteria, prediction scores and summarized results of functional assays.

See excel file Table_S1_S4.

References

1. Madeira, F., Madhusoodanan, N., Lee, J., Eusebi, A., Niewielska, A., Tivey, A.R.N., Lopez, R., and Butcher, S. (2024). The EMBL-EBI Job Dispatcher sequence analysis tools framework in 2024. Nucleic Acids Res *52*, W521–W525. https://doi.org/10.1093/nar/gkae241.
