# Supplementary material for: De novo variants in LDB1 are linked to distinct neurodevelopmental phenotypes determined by variant location and differing pathomechanisms
Source: Am J Hum Genet. 2026 Jun 19;113(7):1543–57. doi: 10.1016/j.ajhg.2026.05.012 (PMC13504347; doi:10.1016/j.ajhg.2026.05.012)
Supplement: Supplementary file 2 [file mmc3.pdf]

ARTICLE

De novo variants in *LDB1* are linked to distinct neurodevelopmental phenotypes determined by variant location and differing pathomechanisms

Graphical abstract

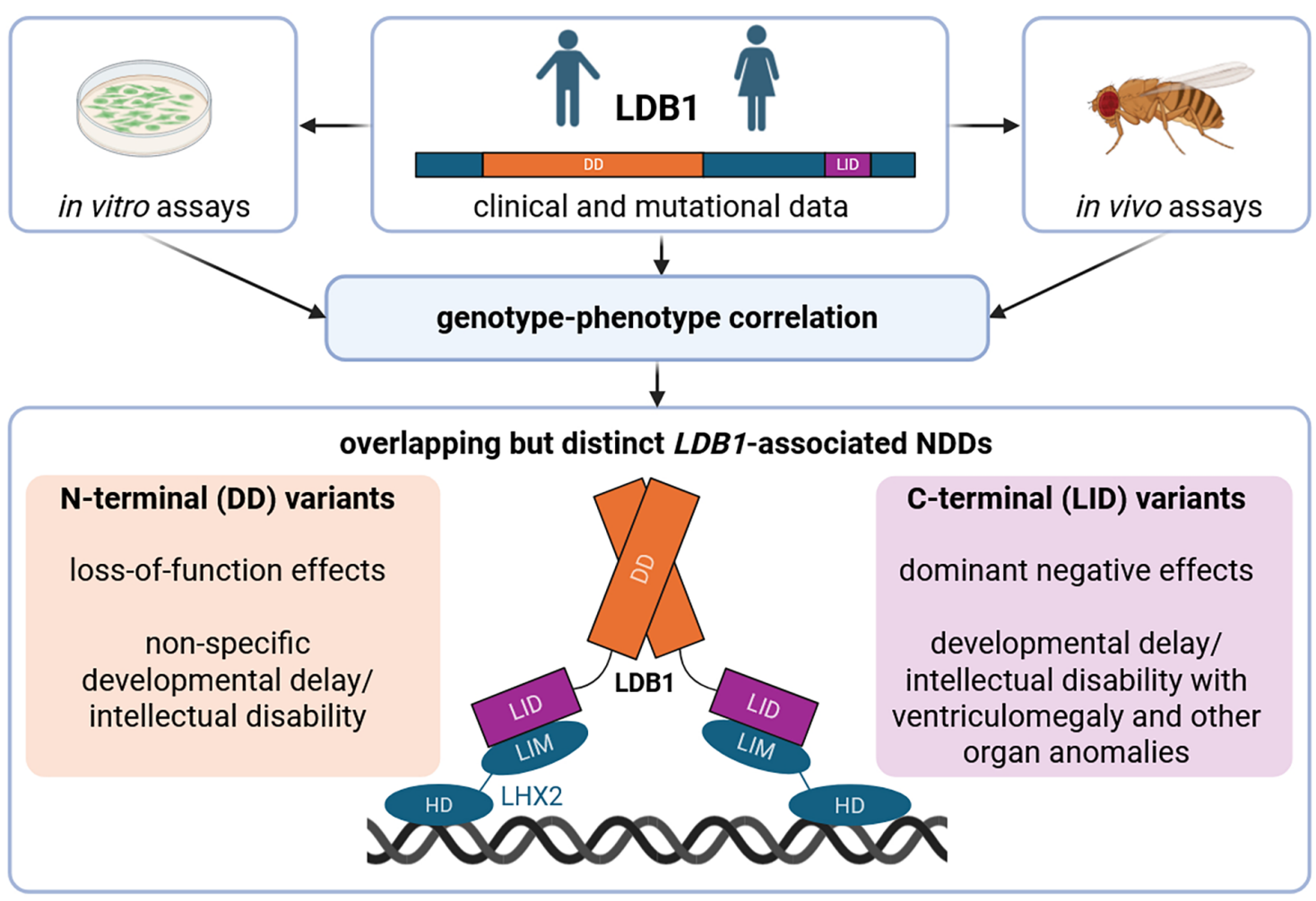

Authors

Rebecca Fluri, Mireia Coll-Tané, Theresa Brunet, ..., Christiane Zweier, Heinrich Sticht, Anne Gregor

Correspondence

anne.gregor@unibe.ch

***De novo* variants in *LDB1* were linked to distinct neurodevelopmental disorders (NDDs). N-terminal missense and truncating variants lead to loss of function and variable NDDs without brain anomalies, and C-terminal missense or truncating variants act through a dominant-negative mechanism and lead to NDDs with ventriculomegaly and organ anomalies.**

Fluri et al., 2026, The American Journal of Human Genetics *113*, 1543–1557
July 2, 2026

© 2026 The Author(s). Published by Elsevier Inc. on behalf of American Society of Human Genetics.

https://doi.org/10.1016/j.ajhg.2026.05.012

CellPress

De novo variants in *LDB1* are linked to distinct neurodevelopmental phenotypes determined by variant location and differing pathomechanisms

Rebecca Fluri,[1,2] Mireia Coll-Tané,[3,4] Theresa Brunet,[5] Benjamin Cogne,[6,7] Solene Conrad,[6] Mathilde Nizon,[6] Francesco Nicita,[8] Lorena Travaglini,[9] Antonio Novelli,[9] Margie Glissmeyer,[10] Amanda Peterson,[10,11] Jillian G. Buchan,[12] Dan Serber,[12] Kolja Meier,[13] Jutta Gärtner,[13] Susann Diegmann,[13] Veronique Pingault,[14] Tania Attie-Bitach,[14] Thomas Courtin,[15] Michael C. Schneider,[16] Wing Hung,[16] Inderneel Sahai,[17] Lauren O'Grady,[17] Katharina Steindl,[18] Sarju G. Mehta,[19] Christel Depienne,[20] Delphine Heron,[21] Boris Keren,[21] Solveig Heide,[21] Shane McKee,[22] Franco Laccone,[23] Lisa M. Dyer,[24] Catherine Melver,[25] Connie Motter,[25] Wendy D. Jones,[26] Zoey Trueblood Wilson,[27] Divya Vats,[27] Kristina Huß,[28] Christiane Zweier,[1,2] Heinrich Sticht,[29] and Anne Gregor[1,2,*]

Summary

LDB1 encodes transcriptional regulator protein LIM domain-binding protein 1, which plays an important role in neurogenesis. Few C-terminal likely gene-disrupting (LGD) variants have been reported in the literature in individuals with congenital ventriculomegaly. Through international collaboration, we now assembled a cohort of 16 individuals with *de novo* variants affecting various regions of *LDB1*. Eleven variants affect either the whole gene or the N-terminal dimerization domain (including gene deletions, as well as nonsense-mediated mRNA decay (NMD)-sensitive LGD and missense variants), and five variants (missense or NMD-escaping LGD variants) affect only the C terminus of LDB1 containing the LIM interaction domain. All individuals showed variable neurodevelopmental phenotypes, including developmental delay and behavioral anomalies. In line with literature reports, individuals harboring C-terminal variants additionally presented with ventriculomegaly, which suggests a potential genotype-phenotype correlation. In accordance, we found diverging pathomechanisms *in vitro*: N-terminal missense variants disrupt homodimerization of LDB1, likely leading to a loss of function, while C-terminal variants impair interaction with the essential partner LHX2 in a dominant-negative manner. These findings were confirmed *in vivo* in *Drosophila melanogaster*. Toxicity of overexpressed human LDB1 in *Drosophila* was not observed with N-terminal missense variants but was exacerbated by C-terminal variants. Similarly, phenotypes associated with *LDB1/chi* loss were rescued by overexpression of wild-type LDB1 but not by LDB1 harboring N-terminal missense variants or by C-terminal variants that even worsened phenotypes. In summary, our findings indicate that *de novo* variants in *LDB1* are linked to two overlapping but distinct neurodevelopmental phenotypes based on variant location and propose two separate pathomechanisms underlying *LDB1*-related neurodevelopmental disorders.

[1]Department of Human Genetics, Inselspital University Hospital Bern, University of Bern, 3010 Bern, Switzerland; [2]Department for Biomedical Research (DBMR), University of Bern, 3010 Bern, Switzerland; [3]Department of Human Genetics, Radboud University Medical Center, 6525 GA Nijmegen, the Netherlands; [4]Donders Institute for Brain, Cognition and Behaviour, Radboud University Medical Center, 6525 GA Nijmegen, the Netherlands; [5]TUM School of Medicine and Health, Institute of Human Genetics, TUM University Hospital, Technical University of Munich, 81675 Munich, Germany; [6]Nantes Université, CHU de Nantes, Service de Génétique Médicale, 44000 Nantes, France; [7]Nantes Université, CHU de Nantes, CNRS, INSERM, L'institut du Thorax, 44000 Nantes, France; [8]Unit of Muscular and Neurodegenerative Diseases, Bambino Gesù Children's Hospital, IRCCS, 00165 Rome, Italy; [9]Laboratory of Medical Genetics, Translational Cytogenomics Research Unit, Bambino Gesù Children's Hospital, IRCCS, 00165 Rome, Italy; [10]Seattle Children's Hospital, Seattle, WA 98105, USA; [11]Department of Molecular and Medical Genetics, Oregon Health and Science University, Portland, OR 97239, USA; [12]Department of Laboratory Medicine and Pathology, University of Washington, Seattle, WA 98195, USA; [13]Department of Pediatrics and Adolescent Medicine, Division Pediatric Neurology and German Center for Child and Adolescent Health (DZKJ), University Medical Center Göttingen, 37075 Göttingen, Germany; [14]Service de Médecine Génomique des Maladies Rares, AP-HP Centre, Hôpital Necker-Enfants Malades, 75015 Paris, France; [15]Center for Molecular and Chromosomal Genetics, AP-HP-Sorbonne University, Pitié-Salpêtrière Hospital, 75013 Paris, France; [16]Section of Neurology, Department of Pediatrics, St. Christopher's Hospital for Children, Drexel University College of Medicine, Philadelphia, PA 19134, USA; [17]Division of Medical Genetics and Metabolism, Massachusetts General Hospital for Children, Boston, MA 02114, USA; [18]Institute of Medical Genetics, University of Zurich, 8952 Zurich, Switzerland; [19]Department of Clinical Genetics, Cambridge University Hospitals NHS Foundation Trust, and University of Cambridge, Cambridge CB2 0QQ, UK; [20]Institute of Human Genetics, University Hospital Essen, University of Duisburg-Essen, 45122 Essen, Germany; [21]Département de Génétique, AP-HP-Sorbonne Université, Hôpital Trousseau & Groupe Hospitalier Pitié-Salpêtrière, 75013 Paris, France; [22]Northern Ireland Regional Genetics Service, Belfast City Hospital, Belfast BT9 7AB, UK; [23]Center for Pathobiochemistry and Genetics, Institute of Medical Genetics, Medical University of Vienna, 1090 Vienna, Austria; [24]GeneDx, LLC, Gaithersburg, MD 20877, USA; [25]Division of Medical Genetics, Akron Children's Hospital, Akron, OH 44302, USA; [26]The North East Thames Regional Genetics Service, Great Ormond Street Hospital, London WC1N 3JH, UK; [27]Southern California Kaiser Permanente Regional Metabolic Genetics Center, Los Angeles, CA 90041, USA; [28]LMU Klinikum, Campus Innenstadt, Dr. von Haunersches Kinderspital und iSPZ Hauner MUC, 80337 München, Germany; [29]Institut für Biochemie, Friedrich-Alexander-Universität Erlangen-Nürnberg, 91054 Erlangen, Germany

*Correspondence: anne.gregor@unibe.ch

https://doi.org/10.1016/j.ajhg.2026.05.012.

© 2026 The Author(s). Published by Elsevier Inc. on behalf of American Society of Human Genetics.
This is an open access article under the CC BY license (http://creativecommons.org/licenses/by/4.0/).

Introduction

LIM domain binding gene 1 (*LDB1*; also known as *NLI* and *CLIM2*) (MIM: 603451) encodes a ubiquitously expressed co-regulator of transcription.[1] Studies in mice have shown its importance in neurogenesis, including development of the fore-,[1] mid- and hindbrain.[2] Additionally, it plays an essential role in the differentiation of corticospinal motoneurons and interneurons,[3] axon guidance,[4] retinal gliogenesis,[5] and expression of receptors in olfactory sensory neurons.[6] Its effects outside the nervous system are diverse and notably include regulation of hematopoiesis, cardiogenesis, and metabolic functions in hepatocytes and pancreatic cells.[7]

LDB1 itself is a non-DNA-binding protein that regulates transcription through the formation of multimeric protein complexes. Depending on its interaction partners, it can act as a transcriptional activator or repressor. It has two domains that are essential for protein-protein interactions: the N-terminal dimerization domain (DD) enables homodimerization and the C-terminal LIM interaction domain (LID) facilitates heterodimerization with LIM proteins, including LIM domain only (LMO) and LIM homeobox (LHX) proteins.[8,9] LIM proteins play an important role in embryonic development and cell type determination. Disruptions of various LHX proteins in different models have revealed their importance specifically in neuronal development.[8] *De novo* variants in the partner protein *LHX2* (MIM: 603759) have recently been identified to cause a neurodevelopmental disorder (NDD),[10] with one of the described disease mechanisms being an impaired interaction capability with LDB1.[10] Very recently, *LDB1* has also been tentatively linked to an NDD. Eight C-terminal likely gene disrupting (LGD) variants and one missense variant have been described in individuals with an NDD mainly characterized by congenital ventriculomegaly.[11–13] Other, less consistently observed clinical features included global developmental delay, dysmorphic features and autism in some cases. However, individual-level clinical data are missing for the majority of cases.[11–13]

To better understand the clinical presentation of *LDB1*-associated NDDs, we established a cohort of 16 individuals with variable neurodevelopmental phenotypes harboring previously unreported missense and LGD variants distributed across *LDB1*. In functional assays, we observed alterations in protein expression levels and impaired protein-protein interactions depending on variant location. Rescue models and overexpression of LDB1 variants in *Drosophila melanogaster* further corroborated different pathomechanisms based on variant location, indicating a loss-of-function effect for N-terminal variants and a dominant-negative effect for C-terminal variants.

Subjects and methods

Subjects

Through personal communication, GeneMatcher,[14] and DECIPHER (Database of Chromosomal Imbalance and Phenotype in Humans using Ensembl Resources),[15] information on 16 individuals with variants in *LDB1* was assembled. Clinical data were collected using a standardized Excel spreadsheet (Table S1; pedigrees are shown in Figure S1). Testing by chromosomal microarray or exome or genome sequencing was done either in a diagnostic setting without the need for specific ethics approval ("clinical") or in a research setting with ethics approval from the respective review boards (Table S1). Variants were annotated according to the MANE (Matched Annotation from NCBI and EMBL-EBI) select transcript (GenBank: NM_001113407.3; GenBank: NP_001106878.1) and preliminarily classified according to ACMG (American College of Medical Genetics and Genomics) criteria[16] plus current ClinGen sequence variant interpretation recommendations (https://clinicalgenome.org/tools/clingen-variant-classification-guidance/). The criteria application and variant classification presented in this paper do not necessarily reflect the criteria and classification of the clinical testing laboratories involved in this paper.

The individuals or their parents or legal guardians have given informed consent for publication of the data and pictures. The study complied with the principles set out in the Declaration of Helsinki.

In silico analysis

Multiple sequence alignment for conservation analysis of missense variants was done using Clustalw2.[17] Pathogenicity for missense variants was assessed using different prediction tools: CADD,[18] REVEL,[19] SIFT,[20] AlphaMissense,[21] and Primate AI.[22] Potential effects on splicing were assessed using SpliceAI.[23] Disorderedness of C-terminal LID-affecting variants was assessed using PONDR (https://www.pondr.com/). Charge analysis was performed using the EMBOSS charge tool with a window size of 8 (https://www.bioinformatics.nl/cgi-bin/emboss/charge). Isoelectric points (pI) of wild-type and mutated proteins were calculated using the Expasy compute pI tool (https://web.expasy.org/compute_pi/).

Variants c.361C>T (GenBank: NM_001113407.3) (p.Arg121Trp), c.542G>A (p.Arg181Gln), and c.577C>T (p.Arg193Trp) were modeled based on the crystal structure of the LDB1 DD (PDB: 8HIB;[24]). For interpretation of the c.1075A>C (p.Thr359Pro) variant, the LDB1-LHX2 complex was modeled using the structure of the homologous LDB1-LHX3 complex (PDB: 2JTN)[25] as a template. Variant residues were introduced in the wild-type structures using SwissModel,[26] and RasMol[27] was used for structure analysis and visualization.

Plasmids

Expression constructs containing N-terminally FLAG- or hemagglutinin (HA)-tagged *LDB1* (isoform: GenBank: NM_001113407.3) were subcloned into the pcDNA3.1 vector. Four missense variants identified in this study (p.Arg121Trp, p.Arg181Gln, p.Arg193Trp, and p.Thr359Pro) and six C-terminal LID-disrupting variants reported here or previously (this study: c.951del [p.Ser317Argfs*21], c.997C>T [p.Gln333*], and c.1068del [p.Arg356Serfs*127]; previously reported: c.906_907del [p.Gly304Glnfs*22], c.957_960del [p.Ser319Argfs*18], and c.1045del

[p.Glu349Serfs*134]) were introduced into the FLAG-LDB1 vector via site-directed mutagenesis using a modified version of the Quikchange site-directed mutagenesis kit (Stratagene, Agilent, Santa Clara, CA, USA). A construct containing Myc-tagged *LHX2* (isoform: GenBank: NM_004789.4) was generated previously.[10]

Protein analysis

For protein analysis, HEK239 cells were transiently transfected with FLAG-tagged wild-type or mutant *LDB1* (1.5 μg plasmid per 6 wells) using the jetPrime system (Polyplus transfection, Illkirch, France). One to two days after transfection, cells were lysed with RIPA buffer (50 mM Tris [pH 8.0], 150 mM NaCl, 1% Igepal CA-630, 0.1% SDS, 0.5% sodium-deoxycholate). To assess protein stability, cells were treated with 25 μM MG132 (Sigma-Aldrich, St. Louis, MO, USA) 24 h post transfection and incubated at 37°C for 4 h prior to lysis.

For SDS-PAGE, protein lysates were mixed with 4× SDS-PAGE sample buffer, separated on a 4%–20% Mini-PROTEAN TGX Stain-Free Gel (Bio-Rad, Hercules, CA, USA), and subsequently blotted onto a nitrocellulose membrane using the semi-dry blotting system (Bio-Rad). Membranes were incubated with the primary antibodies rabbit anti-FLAG (F7425, Sigma, 1:5,000) and rabbit anti-histone H3 (4499; Cell Signaling Technology (CST), Danvers, MA, USA; 1:5,000), followed by incubation with goat anti-rabbit horseradish peroxidase (HRP)-conjugated secondary antibody (170-6515, Bio-Rad, 1:15,000). SuperSignal West Femto Maximum Sensitivity Substrate (Thermo Scientific, Waltham, MA, USA) was used to visualize proteins with the ChemiDoc imaging system (Bio-Rad). Analysis was done with Image Lab software version 6.1 (Bio-Rad). Experiments were carried out at least in triplicates. Protein levels of LDB1 were normalized to the corresponding levels of histone H3, and normalized values were $\log_2$ transformed. Statistical significance was assessed using a one-sample *t* test with the theoretical test value set to 0. An overview of all antibodies used can be found in Table S2.

Immunofluorescence analysis

HeLa cells seeded on coverslips were transfected with wild-type or mutant *LDB1* (500 ng per 12 wells) using the jetPrime system. After 48 h, the cells were fixated with 4% paraformaldehyde in phosphate-buffered saline (PBS) and permeabilized with 0.1% Triton X-100 in PBS. They were stained with primary antibodies mouse anti-FLAG (F1804, Sigma-Aldrich, 1:250) and rabbit anti-nucleolin (14574, CST, 1:100) and the secondary antibodies Alexa Fluor 488 goat anti-rabbit (A11008, Thermo Scientific, 1:500) and Alexa Fluor 546 donkey anti-mouse (A10036, Thermo Scientific, 1:500); nuclei were counterstained with DAPI (Serva, Heidelberg, Germany; 1:50,000). An Axio Imager Z2 with Apotome3 (Carl Zeiss, Oberkochen, Germany) with a 63× objective and Zen software v.3.13 was used for imaging and analysis.

Co-immunoprecipitation

To assess the interaction capability of LDB1 with interaction partners, HEK293 cells were transfected with FLAG-tagged wild-type or mutant *LDB1* (1.5 μg per 6 wells) alongside either Myc-tagged *LHX2* or HA-tagged wild-type *LDB1* (0.5 μg per 6 wells). Additionally, *LDB1* constructs with C-terminal LID-affecting variants were transfected together with wild-type *LDB1* (0.75 μg mutant and 0.75 μg wild-type *LDB1* per 6 wells) and *LHX2*. Two days after transfection, the cells were lysed with RIPA buffer, and the extracted proteins were incubated with anti-FLAG M2 magnetic beads (Sigma-Aldrich), rotating overnight. Beads were washed with RIPA buffer and Tris-buffered saline (TBS), and proteins were subsequently eluted from the beads in 1× SDS-PAGE sample buffer. SDS-PAGE of samples was performed as described for protein analysis above. Primary antibodies rabbit anti-HA (3724, CST, 1:2,000), rabbit anti-Myc (2272S, CST, 1:2,500), and rabbit anti-FLAG (1:5,000) and secondary antibody goat anti-rabbit HRP (1:15,000) were used. Experiments were replicated at least three times. For quantification, immunoprecipitation (IP) of Myc-LHX2 or HA-LDB1, respectively, was normalized to the corresponding IP band(s) of FLAG-LDB1 and $\log_2$ transformed. A one sample *t* test with the theoretical value set to 0 was used to calculate the *p* values.

Drosophila stocks and maintenance

D. melanogaster stocks were raised at room temperature in clear vials containing fly food (agar, yeast, corn flour, sugar, 0.1% methylparaben, and propionic acid) and sealed with mite-proof stoppers. To induce tissue-specific knockdown/overexpression, the upstream activating sequence (UAS)/GAL4 system was used.[28] All crosses were carried out at 28°C to increase knockdown/overexpression efficiency.[29]

Stocks were obtained from the Bloomington Drosophila Stock Center (BDSC; pan-neuronal drivers elav-GAL4/CyO [BL #8765] and elav-GAL4 [BL #8760], glial driver repo-GAL4/Tm3 Sb [BL #7415], ubiquitous drivers actin-GAL4/Tm3 Sb Tb [BL #3954] and actin-GAL4/CyO [BL #4414], motoneuron-specific driver D42-GAL4 [BL #8816], RNAi 2 [BL #31049], RNAi 3 [BL #35435], control 2 [BL #36303], overexpression [OE] chi [BL #67741], control human overexpression [hOE] [BL #24749]) and the Vienna Drosophila Research Center (VDRC; RNAi 1 [vdrc107314/KK], control 1 [vdrc60100], RNAi 4 [vdrc30454], control OE [vdrc60000]) or assembled/re-balanced in house (glial driver repo-GAL4/Tm6 Sb Tb).

To generate UAS lines for OE of human wild-type and variant-containing LDB1, the coding sequence of *LDB1* was cloned into the pUAST-attb vector (DGRC stock 1419; https://dgrc.bio.indiana.edu//stock/1419). Variants observed in our cohort were introduced using site-directed mutagenesis (p.Arg121Trp, p.Arg181Gln, p.Arg193Thr, p.Thr359Pro, p.Ser317Argfs*21). After sequence verification, constructs were sent to FlyORF (Zurich, Switzerland) for injection into the line BDSC #24749 (control hOE) to create transgenic flies. For rescue experiments, double-transgenic flies simultaneously expressing RNAi 1 and either wild-type or mutant *LDB1* were generated using a double-balancer line (Kr/CyO;D/Tm6c Sb Tb). See Table S3 for an overview of all fly lines.

RNA isolation and expression analysis

To assess the knockdown/OE efficiency of *LDB1* and its *Drosophila* ortholog *chi*, crosses with ubiquitous dosage manipulation of *chi/LDB1* using the actin-GAL4/Tm3 Sb Tb driver and respective *chi/LDB1*/control lines were carried out. Non-tubby larvae of the progeny were collected, and RNA was isolated using a modified RNeasy Lipid Tissue Mini Plus Kit (QIAGEN, Germantown, MD, USA), where QIAzol was replaced with TRIzol (Thermo Scientific). For assessment of *LDB1* expression in HEK293 cells, cells were transfected as described above, and RNA was isolated using the RNeasy Mini Plus Kit (QIAGEN).

To perform expression analysis, RNA was converted into cDNA using the Superscript II reverse transcriptase and random

hexamer primers (Thermo Scientific). The RT-qPCR reaction was run on a QuantStudio3 using PowerTrack SYBR Green Mastermix (Thermo Scientific) and two primer pairs to amplify *chi* (F1: 5′-CAACGACCACCCAACAAGAG-3′/R1: 5′-TCGTCCTCCTCACCAAACTC-3′ and F2: 5′-GAACATTCCCGGCAACTACC-3′/R2: 5′-CTGGCCACTGTTAAACGGAG-3′) or a primer pair to amplify *LDB1* (F: 5′-TGTCACGCCACAAGACCTAC-3′/R: 5′-CTGACATCTTCCGTTTCCGC-3′). The subsequent analysis was done using the QuantStudio Design and Analysis software 2.5.1 (Thermo Scientific), and the relative expression of *chi/LDB1* was determined using the ΔΔCT method with tubulin (*Drosophila*) or *B2M* (HEK293 cells) as endogenous control.

Viability analysis

Viability was assessed for fly lines with ubiquitous dosage manipulation of *chi* and fly lines with ubiquitous OE of mutant or wild-type *LDB1* in a *chi*-deficient or wild-type background. Two actin-GAL4 driver lines (actin-GAL4/Tm3 Sb Tb and actin-GAL4/CyO) were used for dosage manipulation. Crosses were set up in duplicate vials, and flies were counted separated by sex and genotype daily for 4–6 days until all balancer flies had hatched. Results were confirmed in at least one additional independent experiment. Statistical significance was determined by comparing the percentage of actin-GAL4 vs. balancer (Tm3 Sb Tb or CyO) flies using a Student's *t* test with Bonferroni correction to account for multiple comparisons.

Negative geotaxis assay

Climbing behavior was assessed using the negative geotaxis assay[30] and performed as described elsewhere.[31] Dosage manipulation was induced either pan-neuronally (elav-GAL4/CyO), motoneuron specifically (D42-GAL4), or in glial cells (repo-GAL4/Tm3 Sb). In brief, flies were collected 0-48 h post eclosion and transferred into vials, each containing five female and five male flies. At least 10 vials with 10 flies (total = 100 flies) were tested per condition/genotype, and the results were validated in at least one independent experiment. After recovery from CO_2 anesthesia for at least 24 h, flies were transferred to test vials with an adjustment time of 1 min. The vials were placed below a light source, and flies were tapped to the bottom of the vial and filmed with a camera (Canon PowerShot, SX620 HS). From the film, the fraction of flies that climbed above the target line at 8 cm after 10 s were measured using the software avidemux. The examiner was blinded to the genotype of the flies during the analysis. Statistical analysis was done using the Wilcoxon signed-rank test in Rstudio (v.2022.07.0x548) with Bonferroni correction to account for multiple comparisons.

Bang sensitivity assay

Seizure susceptibility was assessed using the bang sensitivity assay as previously described.[31] Dosage manipulation was induced either pan-neuronally (elav-GAL4/CyO) or in glial cells (repo-GAL4/Tm3 Sb). Fly collection was done as described for the climbing assay. The assay was performed by vortexing the flies for 10 s and subsequently measuring the fraction of flies undergoing spasms 5 s after being lifted from the vortex (Vortex Genie 2, Scientific Industries). The filming, analysis, and statistical analysis were done with the same tools as the negative geotaxis assay.

Sleep monitoring and analysis

Locomotor activity and sleep were recorded with the Drosophila Activity Monitor (DAM2) system (Trikinetics, Waltham, MA, USA). Dosage manipulation was induced either pan-neuronally (elav-GAL4) or in glial cells (repo-GAL4/Tm6 Sb Tb). In brief, 3- to 5-day-old male flies were individually placed without CO_2 anesthesia in transparent tubes (65×5 mm) containing standard food and loaded into the DAM systems. Flies were allowed to acclimate to activity monitors and food for at least 12 h and were then monitored for 4 days at 25°C in a 12:12 light/dark (LD) cycle. Motion was detected via the monitors' infrared light beams, and sleep (defined in *Drosophila* as 5 or more minutes of inactivity)[32,33] parameters were extracted using the publicly available Sleep and Circadian Analysis MATLAB Program (SCAMP)[34] for MATLAB. The sleep data presented are the average for the 4 days of data acquisition and from at least three independent experiments ($n = 3$) unless specified otherwise.

To assess significance between two genotypes, we used two-tailed unpaired *t* tests for data following a Gaussian distribution or Mann-Whitney tests for non-parametric data. For groups of more than two genotypes, one-way ANOVA with Bonferroni correction for datasets following a normal distribution or Kruskal-Wallis test with Dunn's multiple comparisons for non-parametric data test was performed. Post hoc Bonferroni correction for multiple testing was further applied for the number of tests performed on a dataset per genotype to determine the corrected two-sided significance level. Only *p* values that passed the corrected significance level are indicated in the figures. Statistical analysis was carried out in GraphPad Prism version 10 for Windows (GraphPad Software, San Diego, CA, USA).

Results

LDB1-associated variant spectrum

We assembled a total of 16 variants in *LDB1* (GenBank: NM_001113407.3), including two small chromosomal deletions encompassing *LDB1* and six N-terminal LGD variants, three of which are nonsense variants (c.196C>T [p.Gln66*], c.591G>A [p.Trp197*], and c.607C>T [p.Gln203*]), two frameshift variants (c.404dup [p.Ala136Cysfs*9] and c.[196del]; [199del], p.[Gln66Lysfs*39]; [Thr67Leufs*38]) (both occurred mosaic in one individual, predicted to add up to around 50% of reads), and one canonical splice-site variant (c.352+1G>A). Additionally, we collected four C-terminal LID-disrupting LGD variants (p.Ser317Argfs*21, p.Gln333*, p.Glu349Serfs*134, and p.Arg356Serfs*127), and four missense variants (p.Arg121Trp, p.Arg181Gln, p.Arg193Trp, p.Thr359Pro) (Figure 1A). One of the variants (p.Glu349Serfs*134) has been published previously,[12,13] and we are providing additional clinical details here. All variants with available parental samples for segregation analysis occurred *de novo* (15/15).

Both chromosomal deletions encompass *PPRC1* (MIM: 617462) alongside *LDB1*, with the larger deletion additionally comprising *HPS6* (MIM: 607522) and *ARMH3* (MIM: 620867) and the smaller deletion comprising *NOLC1* (MIM: 602394). Variants in none of these genes are associated with a dominant rare disorder to date, but possible contributory effects

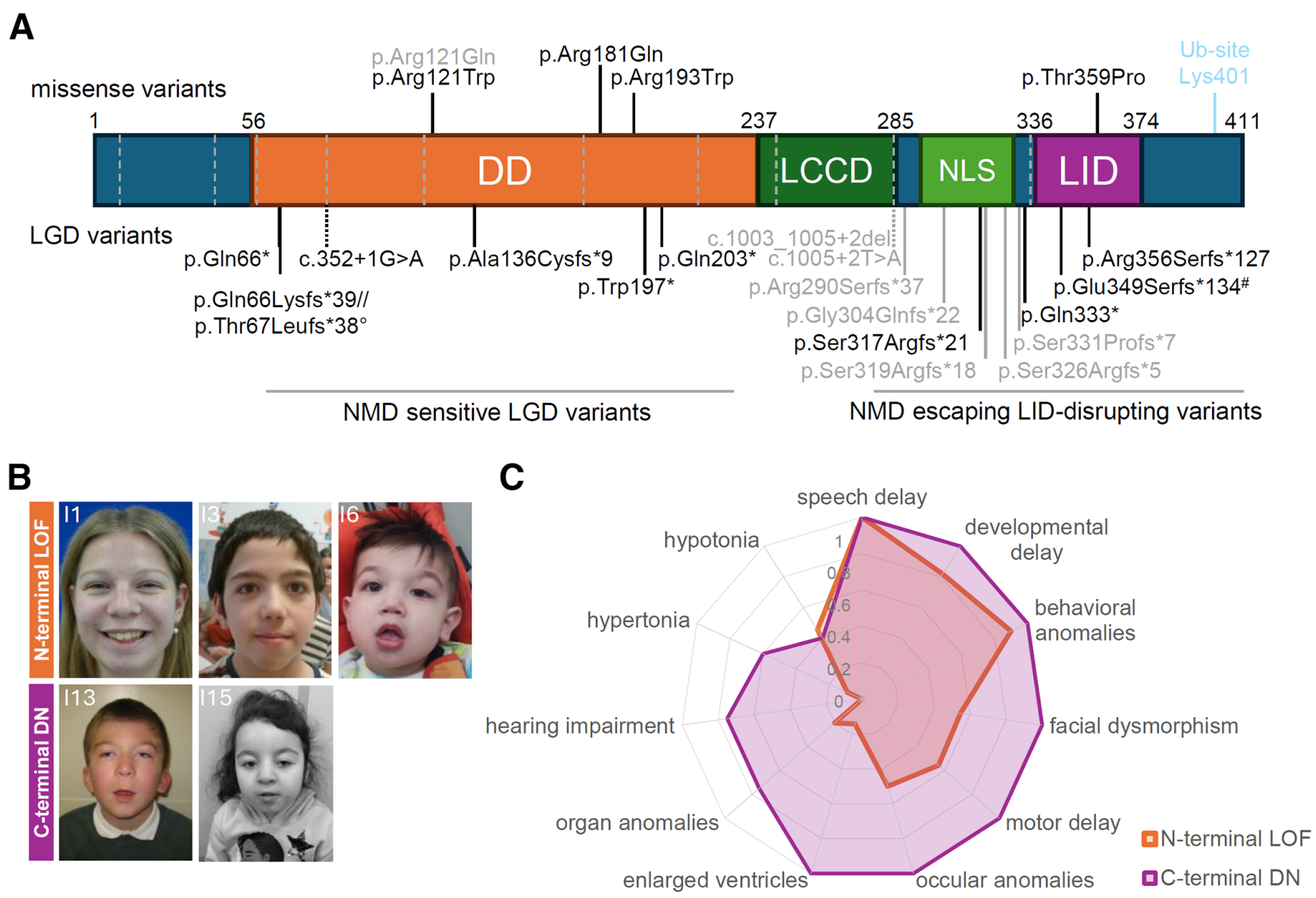

Figure 1. Overview of LDB1 protein structure, identified variants, and clinical images of affected individuals

(A) Schematic of LDB1 (GenBank: NP_001106878.1), with variants assembled in this study labeled in black and previously published variants[11–13] in gray. Of note, nomenclature of variants from Allington et al.[13] was adjusted to correspond with HGSV guidelines. Domains are color coded and identified according to Wang et al.[9] Corresponding exon boundaries are highlighted with gray dashed lines. DD, dimerization domain; LCCD, LDB/chip conserved domain; NLS, nuclear localization signal; LID, LIM interaction domain; Ub, ubiquitination. °Both variants occur in mosaic form in one individual and are predicted to add up to about 50% of reads, correlating with a heterozygous state. #Variant was previously published[12,13] but is included here with additional clinical information on the affected individual.

(B) Clinical images of several affected individuals show facial dysmorphism and highlight similarities between cases.

(C) Phenotypic differences and similarities between newly and previously reported individuals[11–13] harboring N-terminal LGD or DD missense variants (N-terminal loss of function [LoF]) and C-terminal LID-affecting (LID missense, C-terminal frameshift/nonsense/splice site) variants with dominant-negative (DN) effects (C-terminal DN) are shown in a radar plot.

cannot be excluded, especially for the loss of *PPRC1* which is also intolerant to loss-of function variation according to gnomAD v.4.1.0. N-terminal LGD variants are likely to undergo nonsense-mediated mRNA decay (NMD) and subsequently lead to loss of function. In contrast, all C-terminal LGD variants result in a stop codon in the penultimate or last exon and therefore likely escape NMD. These variants are either located shortly before or within the LID of *LDB1*. Most of these C-terminal LID-disrupting variants remove the relatively acidic and disordered C terminus of LDB1 and consequently increase rigidity of the region and the pI of the total protein (Figures S2A–S2C).

Regarding missense variants, one of them (p.Thr359Pro) is also situated in the LID, while the other three reside in the DD. All identified missense variants affect highly conserved amino acids (Figure S2D). Different prediction tools estimate a high chance of pathogenicity for all missense variants (Table S4). The variant p.Arg181Gln, however, is predicted to be less likely deleterious than the others, having the lowest REVEL (0.53) and AlphaMissense (0.59) scores. The LGD variant c.352+1G>A affects a consensus splice site with a high probability of resulting in donor loss (SpliceAI score 0.82), likely leading to skipping of exon 5 and subsequently frameshifting. All variants are very rare and either absent from gnomAD v.4.1.0,[35] or present only in one (p.Gln203*: 1/1610904) or very few (p.Arg181Gln: 4/1613342) individuals.

LDB1 shows strong constraint against loss-of-function (pLI = 1.00, observed/expected = 0.12 [0.07–0.24], LOEUF = 0.24 [loss-of-function observed/expected upper bound fraction]) and missense variation (missense Z score = 3.49, observed/expected = 0.6 [0.55–0.66]) according to population-based metrics from gnomAD v.4.1.0. Furthermore, dosage-sensitivity prediction scores suggest that LDB1 is intolerant to dosage loss (haploinsufficiency; pHaplo = 0.94) and dosage gains (triplosensitivity; pTriplo = 0.90).

When preliminarily classifying *LDB1* variants according to ACMG criteria, all N-terminal LGD variants and C-terminal LID-disrupting variants can, together with our functional data (see below), be classified as likely pathogenic or pathogenic. For the missense variants, three of them can also be classified as likely pathogenic, while the fourth variant, p.Arg181Gln, remains a variant of unknown significance (VUS). We applied the criterion PS3 for all variants on which we performed functional assays, except for p.Arg181Gln, where results did not clearly show deleterious effects (see below). Classification details and criteria used can be found in Table S4.

Clinical spectrum associated with *LDB1* variants

Most affected individuals for whom detailed clinical information was available show developmental delay (14/16) including speech (13/13) and motor delay (8/13), as well as intellectual disability (8/10). The severity in disability ranges from mild to severe, with an inability to speak and walk unaided. Behavioral anomalies (13/14), such as hyperactivity, attentional difficulties, and autism, are also common. MRI anomalies include ventriculomegaly/enlarged ventricles, cerebellar vermis hypoplasia, hypoplastic hippocampi, and partial agenesis of the corpus callosum and facial nerve. Few individuals have been reported having epilepsy (2/13). Organ malformations outside the nervous system occurred rather infrequently and included cardiac defects (3/13), renal anomalies (2/13), and intestinal

Table 1. Summary of available clinical finding in individuals with LDB1 variants (*n* of this study and published cases [*n* of this study])

Clinical finding	N-terminal LGD + DD missense ($n = 12$ [11])	C-terminal LID affecting ($n = 12$ [5])
Developmental delay	9/11 [9/11]	7/7 [5/5]
Speech delay	10/10 [10/10]	5/5 [3/3]
Behavioral anomalies	9/10 [9/10]	4/4 [4/4]
Facial dysmorphism	6/11 [6/11]	6/7 [5/5]
Epilepsy	2/11 [2/11]	0/2 [0/2]
Ventriculomegaly/enlarged ventricles	1/7 [0/6]	9/9 [2/2]
Other organ malformations (cardiac, renal, GI)	2/10 [2/10]	3/4 [2/3]
Hypotonia	5/11 [5/11]	2/5 [1/3]
Hypertonia	1/11 [1/11]	3/5 [2/3]
Motor delay	5/9 [5/9] (mostly mild)	5/5 [3/3]
Vision impairment	5/10 [5/10]	4/4 [4/4]
Hearing loss	0/10 [0/10]	3/4 [3/4]

Published cases[11–13]; GI, gastrointestinal tract.

anomalies (1/8). Variable urogenital anomalies occurred in more than half of the cases (7/13). Choanal atresia was observed in two individuals. Hearing loss (3/14) and ocular abnormalities (9/14), such as cortical visual impairment, coloboma of the optic nerve, ptosis, hyperopia, and strabismus, have also been observed. Minor variable skeletal anomalies are frequently reported (11/16). Non-specific facial dysmorphisms are often noted (11/16), and some individuals share characteristic features (Figure 1B). Growth parameters were not consistently altered and normal for many individuals (abnormal parameters for height, weight, or head circumference in 7/15). An overview of clinical findings can be found in Table 1, and detailed clinical information for all individuals can be found in Table S1.

Genotype-phenotype correlations in individuals with *LDB1* variants

When combining data from this cohort with the sparse available data from published cases, tentative genotype-phenotype correlations can be established. As the number of cases is still rather limited, no statistical validation can be performed, and the results presented are preliminary. It remains to be seen whether the observed genotype-phenotype correlations will hold true in a larger cohort of individuals with additional clinical data. Ventriculomegaly is found in all individuals with detailed clinical information and C-terminal LID-disrupting (C-terminal frameshift, nonsense, and splice site) variants but only in one previously published individual with a missense variant in the DD region. Additionally, severe motor delay, hearing loss, eye abnormalities, facial dysmorphism, and involvement of non-neuronal organs (e.g., heart, kidneys, GI tract) tend to be more prevalent in individuals with C-terminal LID-affecting variants (LID missense and C-terminal LID-disrupting variants). Interestingly, if affected muscle tonus is altered, then it seems to be mirrored between different mutation types. Muscular hypotonia appears to be slightly more prevalent in individuals with N-terminal LGD or missense variants, while hypertonia is predominantly present in individuals with C-terminal LID-affecting variants (Figure 1C). Within the group of N-terminal LGD variants, no phenotypic differences between deletion and point mutation carriers were observed. A summary of phenotypic differences and similarities between variant groups can be found in Table 1.

Mutational modeling

Structural analysis of the DD revealed that the arginine residues 121, 181, and 193 all form stabilizing polar interactions within the DD (Figures 2A–2C and 2E). In the p.Arg121Trp and p.Arg193Trp variants, these polar interactions are completely disrupted by the aromatic tryptophan side chain (Figures 2B and 2F). Therefore, these variants are predicted to cause severe destabilization of the DD, which is also likely to affect its dimerization properties.

In the p.Arg181Gln variant, a salt bridge between the charged wild-type residues Arg181 and Asp158 is replaced by a weaker Gln181-Asp158 hydrogen bond (Figures 2C and 2D). Since this variant at least preserves a polar interaction, it is predicted to have a weaker effect on protein structure compared to the variants at position 121 and 193.

The p.Thr359Pro variant is directly located at the interface between the LDB1 LID and LHX2 (Figures 2G and 2H). Thr359 forms stabilizing polar interactions with Leu67 of LHX2. These interactions cannot be formed by the cyclic and nonpolar Pro359, which additionally causes steric clashes with Leu67. Therefore, this variant is predicted to destabilize the LDB1-LHX2 interface.

Altered LDB1 protein levels of missense and C-terminal LID-disrupting variants

To gain a better understanding of the functional effects of variants in *LDB1*, we conducted *in vitro* experiments.

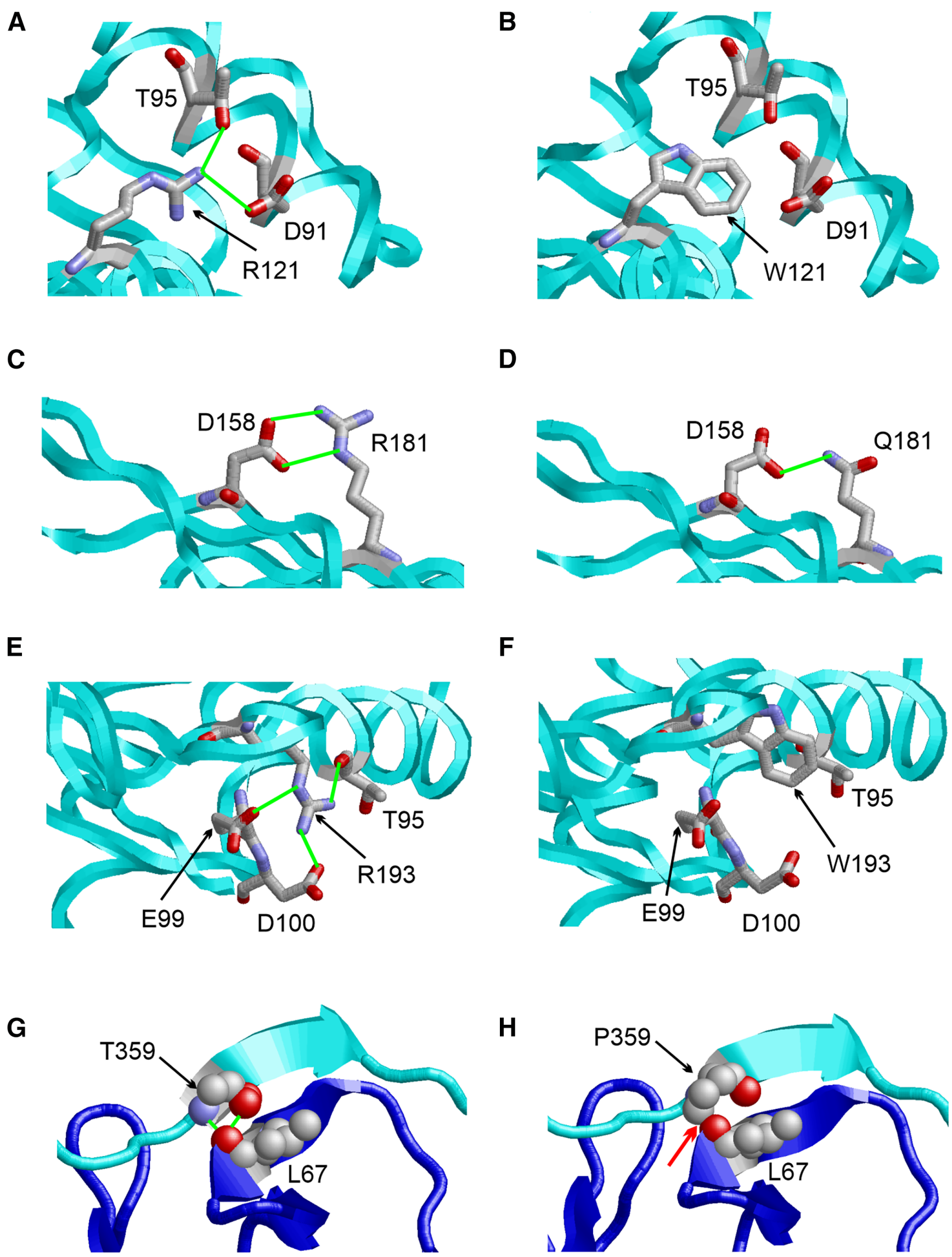

Figure 2. Structural effects of the *LDB1* missense variants
(A) Arg121 of the DD forms polar interactions (green lines) with Asp91 and Thr95. All interacting residues are shown in stick presentation and are colored according to the atom types. The backbone topology of LDB1 is shown as a cyan ribbon.
(B) The aromatic side chain of variant Trp121 cannot form stabilizing interactions with Asp91 or Thr95.
(C) The charged Arg181 forms a salt bridge (green lines) with Asp158.
(D) The polar but uncharged Gln181 exhibits a reduced interaction with Asp158.
(E) Arg193 forms polar interactions (green lines) with Thr95, Glu99, and Asp100.
(F) The aromatic side chain of variant Trp193 cannot form stabilizing interactions with Thr95, Glu99, or Asp100.
(G) Thr359 of the LID forms polar interactions (green lines) with Leu67 of LHX2 (blue). The interacting residues are shown in ball-and-stick presentation and colored according to the atom types. The backbone of LDB1 and LHX2 is shown as a cyan and blue ribbon, respectively.
(H) The variant Pro359 cannot form these polar interactions and causes a steric clash (red arrow) with Leu67 instead.

All published missense and C-terminal LID-disrupting variants reported here and several published previously (Figure 3A) were included in the cellular experiments.

Using transient OE constructs, we investigated the protein levels of wild-type and altered LDB1 in HEK293 cells. *LDB1* transcript levels were comparable between different transfected constructs and not correlating with protein levels (Figure S3A). We observed that the C-terminal LID-disrupting frameshift variants (p.Gly304Glnfs*22, p.Ser317Argfs*21, p.Ser319-Argfs*18, p.Glu349Serfs*134, and p.Arg356Serfs*127) significantly increased the protein levels of altered LDB1 compared to wild-type LDB1 (Figure 3B). Interestingly, protein levels were not increased for the C-terminal nonsense variant p.Gln333*. Additionally, the N-terminal DD missense variant p.Arg193Trp significantly decreased the protein levels of LDB1 in all four biological replicates (Figure 3B and S3B). The other missense variants (p.Arg121Trp, p.Arg181Gln, and p.Thr359Pro) did not consistently alter LDB1 protein levels (Figure 3B).

To determine whether the differences in protein levels were caused by altered degradation rates, transfected HEK293 cells were treated with the proteasomal inhibitor MG132 prior to cell lysis. Protein levels of wild-type LDB1 and LDB1 harboring variants were no longer significantly different following proteasomal inhibition (Figure 3B), suggesting that the varying protein levels were indeed due to increased or reduced degradation.

We additionally analyzed subcellular localization of wild-type LDB1 and LDB1 containing variants using immunofluorescence analysis. Wild-type LDB1 and LDB1 carrying any of the tested missense variants were diffusely distributed in the nucleus (Figure S4). C-terminal frameshift variants, but not the C-terminal nonsense variant p.Gln333*, resulted in the formation of punctate non-nucleolar aggregates in the nucleus (Figure S5).

Impaired interaction capability of LDB1 protein variants with partner proteins

Furthermore, we tested whether variants in LDB1 affect the interaction capability of LDB1 with itself and essential partner proteins. To assess the effect of the variants on the homodimerization potential of LDB1,

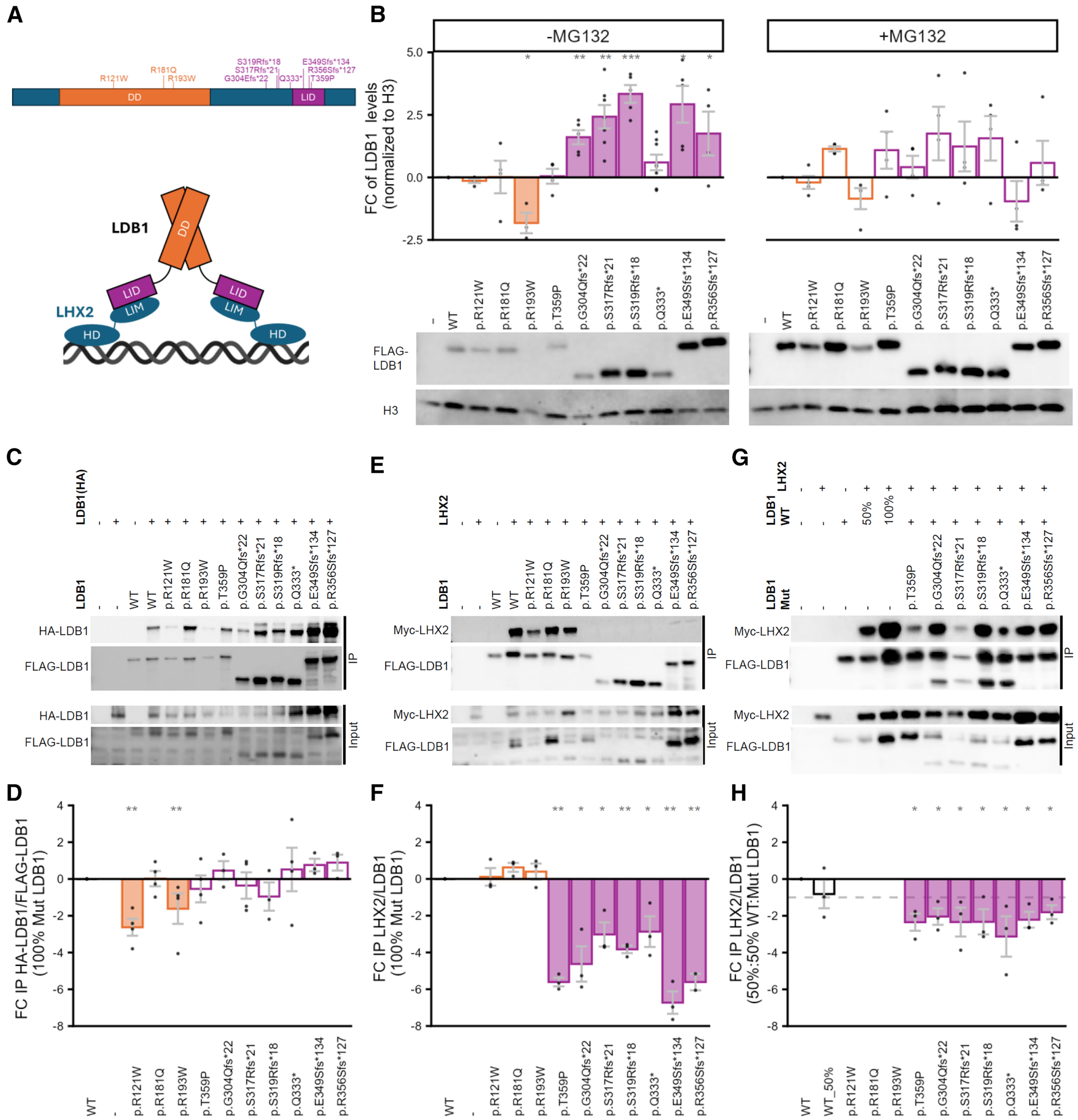

Figure 3. *LDB1* variants affect expression levels and interaction with essential interaction partners
(A) Schematic overview of *LDB1* variants tested in cellular assays and schematic of LDB1-LHX2 tetramer formation together with DNA. Variants are color coded according to the domain they affect (orange, DD purple, LID).
(B) Expression analysis of *LDB1* transiently transfected in HEK293 cells with (+MG132) and without (–MG132) treatment with the proteasomal inhibitor MG132. Without MG132 treatment, reduced expression levels were found for p.Arg193Trp and increased expression levels for several C-terminal frameshift variants. A representative image of the western blot and quantification from at least three independent experiments are shown. LDB1 protein levels were normalized to H3.
(C–H) Co-immunoprecipitation (coIP) of FLAG-tagged wild-type or altered LDB1 with HA-tagged wild-type LDB1 (C and D) or Myc-tagged LHX2 (E–H) showed impaired homodimerization abilities for two DD missense variants and impaired interaction with LHX2 for all C-terminal LID-affecting variants. For interaction with LHX2, simultaneous OE of wild-type LDB1 and LDB1 protein variants indicated DN effects of tested variants (G and H). Representative images (C, E, and G) and quantifications from at least three independent experiments (D, F, and H) are shown. The gray dashed line in (H) represents the expected value for 50% wild-type input. For all experiments here (B–H), for quantification, IP of HA-LDB1/Myc-LHX2 was normalized to IP of FLAG-LDB1. Statistical significance was determined using a 1-sample Student's *t* test with a theoretical value of 0 and compared to the LDB1 wild type (WT) (B-F) or LDB1 WT_50% (H). Significant expression changes are highlighted with filled bars ($p < 0.05$) and marked by asterisks (*$p < 0.05$, **$p < 0.01$, ***$p < 0.001$). For visualization, normalized protein level values were $\log_2$ transformed. Individual values are shown as black dots.

coIP of FLAG-tagged LDB1 and HA-tagged wild-type LDB1 was performed. Two of the three missense variants (p. Arg121Trp and p.Arg193Trp) located in the DD of LDB1, impaired homodimerization, suggesting a loss-of-function mechanism. All variants solely affecting the LID did not impair homodimerization (Figures 3C and 3D).

To test the interaction capability of LDB1 protein variants with LIM proteins, coIP of LDB1 with the LIM

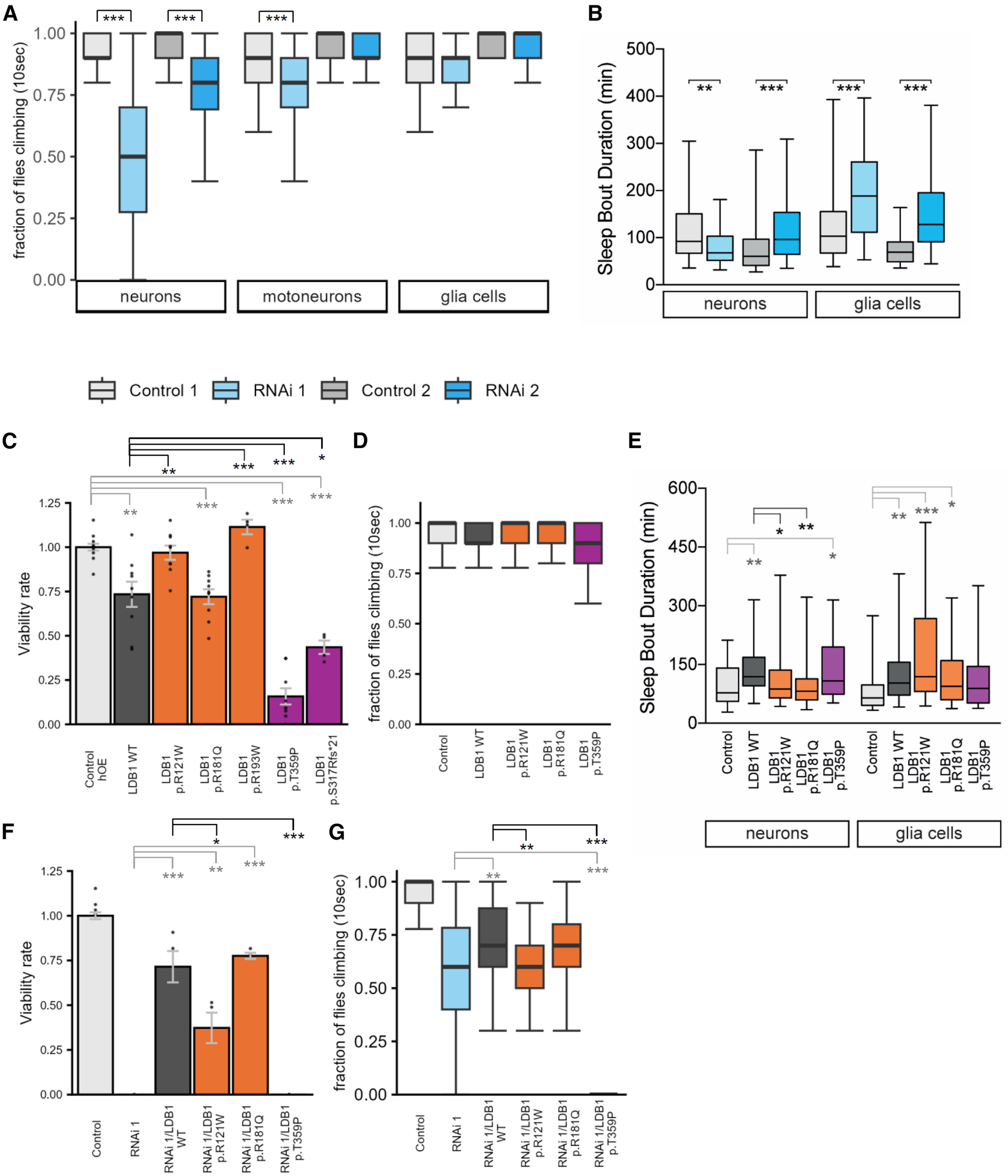

Figure 4. *LDB1/chi* dosage affects viability, negative geotaxis, and sleep behavior in *D. melanogaster*

(A) Negative geotaxis assay showed impaired climbing ability upon pan-neuronal knockdown for two lines and upon motoneuron-specific knockdown of *chi* for one line. Climbing ability was unaffected upon glial knockdown. The fraction of flies climbing at least 8 cm in 10 s per vial (10 flies per vial) was measured. At least 200 flies in batches of 10 ($n = 20$) were tested per genotype. Significance was calculated using a Wilcoxon rank-sum test (***$p < 0.001$).

(B) Average sleep bout duration during the dark period (ZT12–ZT24, [Zeitgeber Time]). Pan-neuronal *chi* knockdown males showed altered sleep bout duration compared to isogenic controls. Pan-glial *chi* knockdown males exhibited longer sleep bouts than their respective controls. At least 60 flies were tested per genotype. Mann-Whitney or ANOVA tests with Bonferroni correction for multiple comparisons were performed for statistics.

(C) Viability of male flies upon ubiquitous OE (using actin-GAL4/Tm3 Sb Tb) of human WT or variant-containing LDB1 was reduced for WT and p.Arg181Gln and even more strongly reduced for variants p.Thr359Pro and p.Ser317Argfs*21. Viability was normalized to the control line, which was set to 1. The experiment was carried out independently at least three times in duplicates each. At least 100 flies were counted per experiment and genotype. Significance was calculated on the ratios of non-Sb (OE) vs. Sb (balancer) flies using a Student's *t* test with Bonferroni correction for multiple testing.

(D) Climbing ability assessed with the negative geotaxis assay was not significantly altered upon pan-neuronal OE of WT or variant-containing LDB1. At least 20 vials were tested per genotype. Significance was determined using a Wilcoxon rank-sum test.

(E) Average sleep bout duration during the dark period (ZT12–ZT24). Neuronal OE of WT LDB1 resulted in longer sleep bouts compared to controls. Pan-neuronal OE of LDB1 variants p.Arg121Trp and p.Arg181Gln significantly shortened sleep bouts compared to WT OE. Glial OE of WT LDB1 and variants p.Arg121Trp and p.Arg181Gln led to longer sleep bouts compared to controls. At least 40–70 flies

(legend continued on next page)

homeobox 2 (LHX2) protein was performed. We observed that the missense variant p.Thr359Pro located in the LID and C-terminal LID-disrupting variants almost completely abolished the interaction of LDB1 with LHX2. The other variants located in the DD did not influence the interaction with LHX2 (Figures 3E and 3F).

As variants are present in a heterozygous state in affected individuals, we wanted to assess whether this interaction was impaired in a dominant-negative manner. To test our hypothesis, we co-transfected wild-type LDB1 at an equimolar ratio with LDB1 variants and with LHX2. We observed that co-transfection of LDB1 containing one of the C-terminal LID-disrupting variants or missense variant p.Thr359Pro negatively affects the heterodimerization of wild-type LDB1 with LHX2, confirming our hypothesis that these variants act in a dominant-negative manner (Figures 3G and 3H).

Confirming dosage sensitivity of *LDB1* ortholog *chi* in *D. melanogaster*

To gain a better understanding of the importance of *LDB1* in development, we investigated its function *in vivo* using the model system *D. melanogaster*. In flies, *chi* (chip) is the well-conserved ortholog of human *LDB1* and *LDB2*, with 59% and 56% sequence identity, respectively. Conservation is even higher in DD and LID regions (Figure S6A). Knockdown of the *Drosophila* ortholog is a valuable model for the heterozygous LGD variants in our cohort. Due to the suggested dosage sensitivity of *LDB1*, we tested effects of both knockdown and OE of commercially available RNAi and OE lines. Validation of four different RNAi knockdown lines confirmed a knockdown efficiency to 51%–58% residual levels in two of them (RNAi1 and RNAi2) and 6-fold OE (OE chi) upon ubiquitous dosage manipulation (Figures S6B and S6C). Ubiquitous knockdown of *chi* was lethal, and OE significantly reduced viability, highlighting its importance for development and the dosage sensitivity of *LDB1/chi* (Figures S6E and S6F). RNAi lines 3 and 4 were excluded from further experiments due to insufficient knockdown efficiency and consistent lack of lethality phenotypes (Figure S6E).

To gain more insight into the neurodevelopmental functions of *chi*, we assessed basic locomotor function using the negative geotaxis assay upon dosage manipulation of *chi* in neurons, motoneurons, or glial cells. Knockdown and OE of *chi* in all neurons or specifically in motoneurons resulted in significant locomotion defects (Figures 4A and S7A). Climbing impairment was more severe upon pan-neuronal knockdown compared to motoneurons only and was most severe with RNAi 1. In contrast, glial knockdown did not affect locomotor behavior, while glial OE mildly impaired climbing ability (Figure 4A and S7A). This suggests a more prominent role of *LDB1/chi* in neurons compared to glial cells. Flies did not show any increased seizure susceptibility in the bang sensitivity assay upon neuronal or glial knockdown or OE (Figures S7B and S7C).

Last, we investigated whether *chi* regulates behavior commonly disrupted in NDDs. While sleep disturbances are highly prevalent in these conditions,[36] sleep analysis can additionally be used as a very relevant readout to study the biological relevance of *chi/LDB1* in neural circuitry. Pan-neuronal knockdown of *chi* using either RNAi line significantly altered sleep episode duration (Figure 4B). However, the direction of the effect differed between knockdown with different RNAi lines; RNAi 1 reduced episode length, whereas RNAi 2 increased it. In the latter case, longer episodes occurred less frequently, indicating enhanced sleep consolidation (Figure S8A). Similarly, glial *chi* knockdown with both RNAi lines consistently led to significantly longer sleep episodes (Figure 4B), accompanied by a reduction in the number of sleep bouts (Figure S8A). These changes suggest that pan-glial *chi* knockdown promotes sleep consolidation and a hypersomnia-like phenotype. Taken together, our findings demonstrate that the *Drosophila* ortholog of *LDB1* regulates sleep architecture.

OE of human LDB1 in *D. melanogaster* leads to toxicity

Having observed different functional consequences of variants in *LDB1* on a cellular level, we tested whether we could observe correlating effects *in vivo* by overexpressing LDB1 in *D. melanogaster*. We first confirmed similar expression levels at the mRNA level for wild-type and mutant *LDB1* in third-instar larvae upon ubiquitous OE (Figure S6D) but cannot rule out differences at the protein level. Ubiquitous OE (driver: actin-GAL4/Tm3 Sb Tb) of wild-type human LDB1 resulted in reduced viability, indicating toxicity of the human protein (Figure 4C). In contrast, OE of mutant *LDB1* containing variants

were tested per genotype. For statistical analysis, Mann-Whitney or ANOVA tests with Bonferroni correction for multiple comparisons were done.

(F) Ubiquitous OE of human WT LDB1 in *chi* knockdown flies could rescue the lethality phenotype, as could p.Arg181Gln. Variant p.Arg121Trp could rescue the lethality significantly less efficiently, while p.Thr359Pro could not rescue it at all. The experiment was carried out twice independently in duplicates, each with at least 100 flies counted per genotype and experiment. Significance was calculated on the ratios of non-Sb (OE) vs. Sb (balancer) flies using a Student's *t* test.

(G) Pan-neuronal OE of human WT LDB1 in neuronal *chi* knockdown flies could partially rescue climbing impairment observed in the negative geotaxis assay. Variant p.Arg121Trp was unable to rescue the defect, while p.Thr359Pro significantly worsened the phenotype compared to *chi* knockdown alone. The experiment was carried out twice from independent crosses, and at least $n = 20$ (200 flies in batches of 10) were tested per experiment.

Significance was calculated using a Wilcoxon rank-sum test. $*p < 0.05$, $**p < 0.01$, $***p < 0.001$ (A–G). Bars/boxplots are color coded according to domain effect (orange, DD; purple, LID) (C–G). Data are represented as boxplots (25th to 75th percentiles, median line; whiskers indicate 5th to 95th percentiles) (A, B, D, E, and G) or bar graphs with individual values shown as black dots (C and F).

p.Arg121Trp or p.Arg193Trp did not impair viability. The lack of toxicity suggests that the produced proteins are not functional. Therefore, their OE does not lead to an effect, as opposed to OE of wild-type *LDB1*, indicating a loss-of-function effect for both variants. OE of *LDB1* with the third DD variant p.Arg181Gln impaired viability similar to wild-type LDB1. Additionally, OE of LDB1 harboring C-terminal LID-affecting variants p.Thr359Pro or p.Ser317Argfs*21 as a representative for all C-terminal frameshifting variants showed increased toxicity compared to the wild-type with an even stronger reduction of viability (Figure 4C). These results were confirmed with a second ubiquitous driver line (actin-GAL4/CyO) (Figure S5G).

We tested a subset of these variants (p.Arg121Trp, p.Arg181Gln, and p.Thr359Pro) in further assays. Pan-neuronal OE of wild-type or mutant *LDB1* did not significantly impair climbing ability in the negative geotaxis assay (Figure 4D). Finally, we assessed the impact of overexpressing *LDB1* with any of these variants in neurons or glial cells on sleep architecture. OE of wild-type human LDB1 in neurons resulted in longer sleep bouts (Figure 4E). In contrast, OE of LDB1 with the variants p.Arg121Trp or p.Arg181Gln significantly shortened sleep episodes compared to wild-type LDB1 (Figure 4E). Notably, only flies overexpressing LDB1 with variant p.Arg181Gln compensated for this sleep loss by increasing the number of sleep episodes (Figure S8B). In contrast, pan-glial OE of wild-type LDB1 or mutant *LDB1* with the variants p.Arg121Trp or p.Arg181Gln significantly increased sleep bout duration (Figure 4E). Interestingly, for p.Arg121Trp, we observed a tendency toward more consolidated sleep compared to wild-type OE.

Rescue of knockdown phenotype in *chi*-deficient *D. melanogaster* with human *LDB1*

Expanding on our previous findings, we wanted to test whether OE of wild-type or mutant human *LDB1* could rescue the *chi* knockdown phenotype observed in *D. melanogaster*. We included three representative variants tested in previous assays in our rescue experiments, reflecting the different proposed mechanisms (loss of function: p.Arg121Trp; dominant negative: p.Thr359Pro; and the VUS p.Arg181Gln). When first looking at ubiquitous knockdown of *chi* (with RNAi 1), additional OE of wild-type *LDB1* rescued the lethality phenotype and significantly increased viability. OE of mutant *LDB1* with the variant p.Arg181Gln was similarly effective. In contrast, OE of *LDB1* with variant p.Arg121Trp increased viability by a significantly lesser degree. Furthermore, OE of *LDB1* with the variant p.Thr359Pro did not rescue the knockdown phenotype, also resulting in complete lethality (Figure 4F).

To further substantiate our results, we also performed rescue experiments for the negative geotaxis assay in pan-neuronally *chi*-deficient flies. We observed that OE of wild-type *LDB1* was partially able to improve the impaired climbing ability caused by *chi* deficiency. A similar but not significant rescue tendency was seen for OE of *LDB1* with the variant p.Arg181Gln. In contrast, OE of mutant *LDB1* with the variant p.Arg121Trp could not rescue the knockdown phenotype, and OE with the variant p.Thr359Pro even worsened the basic locomotion defects (Figure 4G).

Together, these findings upon OE of wild-type and mutant human *LDB1* in control and *chi*-deficient backgrounds support the results from the cellular assays, indicating loss-of-function effects for two of three missense variants from the DD, while variants affecting the LID often lead to more severe phenotypes compatible with dominant-negative effects.

Discussion

While *de novo* variants in *LDB1* have recently been associated with congenital ventriculomegaly, only a small cohort of mainly C-terminal LGD variants has been studied thus far.[12,13] By assembling a cohort of 16 individuals with variants in *LDB1* and variable NDD presentations, 15 of which were previously unpublished, we now further expand the clinical phenotype, propose a potential new genotype-phenotype correlation, and suggest likely diverging pathomechanisms based on variant type and location.

LDB1-associated NDD is highly variable, with prevalent features including global developmental delay ranging from mild to severe, intellectual disability, behavioral anomalies, and non-specific dysmorphic facial features. Congenital ventriculomegaly, the key feature of *LDB1*-associated NDD in the literature,[12,13] is very frequent in carriers of C-terminal LID-affecting variants in individuals here or published previously but absent in the newly described individuals with N-terminal missense or LGD variants, apart from one previously published case of an N-terminal missense variant.[13] Additionally, motor delay, hearing loss, ocular anomalies, and other non-brain-related organ anomalies were also more frequent in individuals with C-terminal LID-disrupting variants. Although the number of individuals in each group is still limited, these observations suggest a possible genotype-phenotype correlation with overlapping but distinct clinical presentations depending on variant type and location. Previous studies have established the importance of LDB1 in embryonic development and neurogenesis. For instance, there is evidence that EMX2 and OTX2 are potential non-LIM partner proteins of LDB1 and involved in development of the choroid plexus alongside LHX2 and LHX5, providing speculative insight into how LDB1 could be linked to ventriculomegaly.[1,37]

In line with the observed genotype-phenotype correlation, we discovered different functional consequences for N-terminal and C-terminal variants *in vitro* and *in vivo*. All tested C-terminal LID-affecting variants (missense and

frameshift/nonsense) impaired heterodimerization of LDB1 with LHX2, and equimolar co-expression of wild-type LDB1 and LDB1 variants showed that the protein-protein interaction is impaired in a dominant-negative fashion regardless of variant-dependent alterations of protein levels. Of note, while OE systems are commonly used to assess functional roles of proteins, they do not necessarily reflect endogenous protein levels or stoichiometry of protein complexes in humans. For this, models derived from affected individuals could provide additional insights. The LDB1-and-LHX2 heterotetramer is critical for various neuronal functions, such as axon guidance and development of the hippocampus.[38] LHX2-associated NDD shares many similarities with LDB1-associated NDD. Both are highly variable and share key features, including developmental delay, intellectual disability, behavioral anomalies, and speech impairment. Motor delay, however, was not commonly observed in LHX2-associated NDD, and extra-neural manifestations are even rarer. The LDB1 LIM-interacting domain is highly conserved across species, and its loss impairs interaction with all LIM proteins.[7] It is therefore likely that the C-terminal variants affect the binding capability of LDB1 with other LHXs and LMOs as well, potentially resulting in additional features. In *in vivo* experiments, OE of *LDB1* with C-terminal variants showed even more toxic effects than wild-type LDB1 OE and was not only unable to rescue lethality or locomotor defects in a *chi*-deficient background but instead led to even worse phenotypes. Fly experiments therefore postulate a toxic effect for the C-terminal variants, which can be explained by the dominant-negative effect observed on a cellular level.

Additionally, C-terminal LID-disrupting frameshift variants resulted in increased protein levels. We speculate that this may be due to an impaired degradation of LDB1 due to loss of the ubiquitination site Lys401.[39,40] A previous study has shown that mutation of the ubiquitination site results in a more stable LDB1 protein.[40] Further supporting this hypothesis, we observed protein levels closer to that of the wild-type after proteasomal inhibition. Increased protein levels could potentially further exacerbate the observed dominant-negative effect for these variants, but this remains to be validated experimentally. Interestingly, these frameshift variants lead to a non-native extended C-terminal tail and resulted in the formation of small nuclear aggregates not seen with OE of wild-type LDB1. As we have not assessed expression of variants from the endogenous locus, it remains elusive whether these aggregates are forming in cells of affected individuals. For similar C-terminal frameshifting variants, a novel mutational mechanism involving phase separation has recently been proposed.[41] As nuclear punctate localization in our study differs from previous findings of nucleolar mislocalization, mutational mechanisms may be slightly different.

For two of three N-terminal missense variants located in the DD (p.Arg121Trp and p.Arg193Trp), we observed impaired homodimerization of LDB1, likely leading to loss of function. LDB1 homodimerization is essential for gene activation by enabling long-range DNA looping. For example, LDB1 activates β-globin transcription in erythroid cells by bridging the distant locus control region (LCR) enhancer and β-globin promotor. The LDB1 DD alone fused with LMO2 can fully rescue transcription in LBD1-depleted murine erythroleukemia cells.[40] Other studies have also shown that LDB1/chi-DD is needed for LDB1 functions, including axon guidance, motoneuronal differentiation, and hippocampal development.[4,42] In addition, the variant p.Arg193Trp also decreases protein levels of LDB1, possibly due to reduced protein stability, as predicted by structural modeling, showcasing a second possible disease mechanism for this variant. In *Drosophila*, OE of LDB1 with any of these variants did not reduce viability or affect sleep patterns and could not sufficiently rescue lethality or geotaxis phenotypes in a *chi*-deficient background. Therefore, both *in vitro* and *in vivo* experiments highly suggest that p.Arg121Trp and p.Arg193Trp are loss-of-function variants. We accordingly considered our experimental results sufficient evidence to apply the PS3 criterion in the ACMG criteria for variant classification for these and the C-terminal LID-affecting variants.

By contrast, we did not observe any functional effects in cellular assays for the third N-terminal missense variant p. Arg181Gln. In *Drosophila*, OE and rescue with mutant *LDB1* carrying this variant also did not indicate functional impairment in the lethality assay. Rescue in the negative geotaxis assay, however, yielded intermediate, not fully conclusive results, indicating a possibly mild impairment of LDB1 function due to this variant. Sleep analysis showed activity levels closer to the control than flies with OE of wild-type LDB1, which would also be indicative of a loss-of-function effect. Since we only observed a mild phenotype in some of the *Drosophila* experiments and no effects *in vitro*, it remains unclear whether this variant is pathogenic. We did not consider the evidence from our functional studies strong enough for this variant to include it in the variant classification, and this variant therefore remains a VUS. This variant notably has the lowest pathogenicity scores of all studied variants, and structural modeling suggested only minor changes to the protein structure due to this variant. Interestingly, the individual harboring this variant is one of two individuals with seizures in our cohort and the only one with severe neonatal-onset therapy-resistant epilepsy.

N-terminal LGD variants were not included in cellular functional assays, as they likely undergo NMD and probably result in loss of function. Heterozygous loss-of-function variants in *LDB1* presumably lead to haploinsufficiency, as the gene is predicted to be dosage sensitive. To confirm our hypothesis, we implemented gene knockdown in *Drosophila*, which is an excellent model for haploinsufficiency, since it reduces gene expression by around 50%. We observed locomotor defects upon

pan-neuronal but not glial knockdown of the *LDB1* ortholog *chi in vivo*, with stronger knockdown resulting in more severe defects, confirming the deletion intolerance of *chi* and highlighting the importance of LDB1/chi in the nervous system. In line with the fact that LDB1 forms a complex with LHX3 and ISL1 that is essential for the differentiation of motoneurons in chick embryos,[43] motoneuronal knockdown of *chi* also caused locomotor defects but to a lesser degree than pan-neuronal knockdown. Sleep architecture was altered upon pan-neuronal knockdown but could be confounded by the flies' impaired locomotor activities. Glial knockdown, however, also consistently increased the duration of sleep bouts, indicating that regulation of sleep is indeed influenced by *chi*. Sleep disturbances are among the most common co-occurring features of NDDs, affecting up to 86% of individuals (compared to ~20% of typically developing children[36]). Due to the lack of detailed data on potential sleep disturbances in our cohort, it currently remains elusive and an area of potential future studies, regardless of whether the suggested role of the *LDB1* ortholog *chi* in sleep regulation can be translated to humans. Our data suggest that *LDB1/chi* is important both in neurons and glia cells but plays a more prominent role in neurons. In summary, the experiments emphasize the importance of LDB1 in the brain and confirm its dosage sensitivity, making it likely that N-terminal LGD and missense variants result in haploinsufficiency.

In conclusion, we refine the spectrum of *LDB1*-associated NDDs by identifying a potential genotype-phenotype correlation with two overlapping but distinct *LDB1*-related disorders with different disease mechanisms. Individuals with C-terminal LID-affecting variants seem to present with a more severe phenotype and congenital ventriculomegaly, and variants potentially act in a dominant-negative way, whereas individuals with N-terminal missense or LGD variants seem to be more mildly affected and do not share this feature, and those variants likely lead to haploinsufficiency either through loss-of-function missense mutations or NMD.

Data and code availability

Data generated or analyzed during this study are included in the manuscript and/or the corresponding supplemental information. All variants were submitted to ClinVar (accession numbers ClinVar: SCV007537448–SCV007537460). Both chromosomal deletions are included in the DECIPHER database (DECIPHER: 382848 and 522748). This paper does not report original code. Generated reagents (plasmids and fly lines) are available from the corresponding author upon request with a completed material transfer agreement.

Acknowledgments

We thank the affected individuals and their families for participation in this study. We thank Pleuni Schreurs for excellent experimental support. A.G. is supported by a young investigator grant from the Bern Center for Precision Medicine. This study makes use of data generated by the DECIPHER community. A full list of centers that contributed to the generation of data is available from https://deciphergenomics.org/about/stats and via email from contact@deciphergenomics.org. DECIPHER is hosted by EMBL-EBI, and funding for the DECIPHER project was provided by the Wellcome Trust (grant WT223718/Z/21/Z). *Drosophila* stocks were obtained from the Bloomington Drosophila Stock Center (NIH P40OD018537) and from the Vienna Drosophila Resource Center (VDRC; www.vdrc.at). Plasmids were obtained from the Drosophila Genomics Resource Center (NIH grant 2P40OD010949) (pUASTattb). F.N. is a member of the European Reference Network for Rare Neurological Diseases – Project ID 739510. C.Z. is supported by a grant from the Swiss National Science Foundation (SNSF; 10001220) and is a member of the European Reference Network on Rare Congenital Malformations and Rare Intellectual Disability ERN-ITHACA, funded by the European Union (grant agreement 101156387). K.M., S.D., and J.G. are members of the German Center for Child and Adolescent Health (DZKJ; project 01GL2402A).

Author contributions

Conceptualization, A.G.; data curation and investigation, R.F., M.C.-T., C.Z., H.S., and A.G.; data collection, all authors; formal analysis, R.F., M.C.-T., H.S., and A.G.; supervision, A.G.; visualization, R.F., M.C.-T., H.S., and A.G.; writing – original draft, R.F., M.C-T., and A.G.; writing – review and editing, all authors.

Declaration of interests

L.M.D. is an employee of and may own stock in GeneDx.

Supplemental information

Supplemental information can be found online at https://doi.org/10.1016/j.ajhg.2026.05.012.

Received: February 26, 2026
Accepted: May 26, 2026
Published: June 19, 2026

References

1. Kinare, V., Pal, S., and Tole, S. (2019). LDB1 Is Required for the Early Development of the Dorsal Telencephalon and the Thalamus. eNeuro *6*. ENEURO.0356. https://doi.org/10.1523/ENEURO.0356-18.2019.
2. Kim, S., Zhao, Y., Lee, J.m., Kim, W.R., Gorivodsky, M., Westphal, H., and Geum, D. (2016). Ldb1 Is Essential for the Development of Isthmic Organizer and Midbrain Dopaminergic Neurons. Stem Cell. Dev. *25*, 986–994. https://doi.org/10.1089/scd.2015.0307.
3. Thaler, J.P., Lee, S.-K., Jurata, L.W., Gill, G.N., and Pfaff, S.L. (2002). LIM Factor Lhx3 Contributes to the Specification of Motor Neuron and Interneuron Identity through Cell-Type-Specific Protein-Protein Interactions. Cell *110*, 237–249. https://doi.org/10.1016/S0092-8674(02)00823-1.
4. van Meyel, D.J., van O'Keefe, D.D., Thor, S., Jurata, L.W., Gill, G.N., and Thomas, J.B. (2000). Chip is an essential cofactor for Apterous in the regulation of axon guidance in

Drosophila. Development *127*, 1823–1831. https://doi.org/10.1242/dev.127.9.1823.

5. de Melo, J., Clark, B.S., Venkataraman, A., Shiau, F., Zibetti, C., and Blackshaw, S. (2018). Ldb1- and Rnf12-dependent regulation of Lhx2 controls the relative balance between neurogenesis and gliogenesis in the retina. Development *145*, dev159970. https://doi.org/10.1242/dev.159970.
6. Monahan, K., Horta, A., and Lomvardas, S. (2019). LHX2- and LDB1-mediated trans interactions regulate olfactory receptor choice. Nature *565*, 448–453. https://doi.org/10.1038/s41586-018-0845-0.
7. Liu, G., and Dean, A. (2019). Enhancer long-range contacts: The multi-adaptor protein LDB1 is the tie that binds. Biochimica et Biophysica Acta (BBA) - Gene Regulatory Mechanisms *1862*, 625–633. https://doi.org/10.1016/j.bbagrm.2019.04.003.
8. Jurata, L.W., Kenny, D.A., and Gill, G.N. (1996). Nuclear LIM interactor, a rhombotin and LIM homeodomain interacting protein, is expressed early in neuronal development. Proc. Natl. Acad. Sci. USA *93*, 11693–11698. https://doi.org/10.1073/pnas.93.21.11693.
9. Wang, H., Kim, J., Wang, Z., Yan, X.-X., Dean, A., and Xu, W. (2020). Crystal structure of human LDB1 in complex with SSBP2. Proc. Natl. Acad. Sci. USA *117*, 1042–1048. https://doi.org/10.1073/pnas.1914181117.
10. Schmid, C.M., Gregor, A., Costain, G., Morel, C.F., Massingham, L., Schwab, J., Quélin, C., Faoucher, M., Kaplan, J., Procopio, R., et al. (2023). LHX2 haploinsufficiency causes a variable neurodevelopmental disorder. Genet. Med. *25*, 100839. https://doi.org/10.1016/j.gim.2023.100839.
11. Jin, S.C., Dong, W., Kundishora, A.J., Panchagnula, S., Moreno-De-Luca, A., Furey, C.G., Allocco, A.A., Walker, R.L., Nelson-Williams, C., Smith, H., et al. (2020). Exome sequencing implicates genetic disruption of prenatal neurogliogenesis in sporadic congenital hydrocephalus. Nat. Med. *26*, 1754–1765. https://doi.org/10.1038/s41591-020-1090-2.
12. Torene, R.I., Guillen Sacoto, M.J., Millan, F., Zhang, Z., McGee, S., Oetjens, M., Heise, E., Chong, K., Sidlow, R., O'Grady, L., et al. (2024). Systematic analysis of variants escaping nonsense-mediated decay uncovers candidate Mendelian diseases. Am. J. Hum. Genet. *111*, 70–81. https://doi.org/10.1016/j.ajhg.2023.11.007.
13. Allington, G., Mehta, N.H., Dennis, E., Mekbib, K.Y., Reeves, B., Kiziltug, E., Chen, S., Zhao, S., Duy, P.Q., Saleh, M., et al. (2025). De novo variants disrupt an LDB1-regulated transcriptional network in congenital ventriculomegaly. Brain *148*, 1817–1828. https://doi.org/10.1093/brain/awae395.
14. Sobreira, N., Schiettecatte, F., Valle, D., and Hamosh, A. (2015). GeneMatcher: A Matching Tool for Connecting Investigators with an Interest in the Same Gene. Hum. Mutat. *36*, 928–930. https://doi.org/10.1002/humu.22844.
15. Foreman, J., Perrett, D., Mazaika, E., Hunt, S.E., Ware, J.S., and Firth, H.V. (2023). Annual Review of Genomics and Human Genetics: DECIPHER: Improving Genetic Diagnosis Through Dynamic Integration of Genomic and Clinical Data. Annu. Rev. Genomics Hum. Genet. *24*, 151–176. https://doi.org/10.1146/annurev-genom-102822-100509.
16. Richards, S., Aziz, N., Bale, S., Bick, D., Das, S., Gastier-Foster, J., Grody, W.W., Hegde, M., Lyon, E., Spector, E., et al. (2015). Standards and guidelines for the interpretation of sequence variants: a joint consensus recommendation of the American College of Medical Genetics and Genomics and the Association for Molecular Pathology. Genet. Med. *17*, 405–424. https://doi.org/10.1038/gim.2015.30.
17. Madeira, F., Madhusoodanan, N., Lee, J., Eusebi, A., Niewielska, A., Tivey, A.R.N., Lopez, R., and Butcher, S. (2024). The EMBL-EBI Job Dispatcher sequence analysis tools framework in 2024. Nucleic Acids Res. *52*, W521–W525. https://doi.org/10.1093/nar/gkae241.
18. Schubach, M., Maass, T., Nazaretyan, L., Röner, S., and Kircher, M. (2024). CADD v1.7: using protein language models, regulatory CNNs and other nucleotide-level scores to improve genome-wide variant predictions. Nucleic Acids Res. *52*, D1143–D1154. https://doi.org/10.1093/nar/gkad989.
19. Ioannidis, N.M., Rothstein, J.H., Pejaver, V., Middha, S., McDonnell, S.K., Baheti, S., Musolf, A., Li, Q., Holzinger, E., Karyadi, D., et al. (2016). REVEL: An Ensemble Method for Predicting the Pathogenicity of Rare Missense Variants. Am. J. Hum. Genet. *99*, 877–885. https://doi.org/10.1016/j.ajhg.2016.08.016.
20. Vaser, R., Adusumalli, S., Leng, S.N., Sikic, M., and Ng, P.C. (2016). SIFT missense predictions for genomes. Nat. Protoc. *11*, 1–9. https://doi.org/10.1038/nprot.2015.123.
21. Tordai, H., Torres, O., Csepi, M., Padányi, R., Lukács, G.L., and Hegedűs, T. (2024). Analysis of AlphaMissense data in different protein groups and structural context. Sci. Data *11*, 495. https://doi.org/10.1038/s41597-024-03327-8.
22. Sundaram, L., Gao, H., Padigepati, S.R., McRae, J.F., Li, Y., Kosmicki, J.A., Fritzilas, N., Hakenberg, J., Dutta, A., Shon, J., et al. (2018). Predicting the clinical impact of human mutation with deep neural networks. Nat. Genet. *50*, 1161–1170. https://doi.org/10.1038/s41588-018-0167-z.
23. Jaganathan, K., Kyriazopoulou Panagiotopoulou, S., McRae, J.F., Darbandi, S.F., Knowles, D., Li, Y.I., Kosmicki, J.A., Arbelaez, J., Cui, W., Schwartz, G.B., et al. (2019). Predicting Splicing from Primary Sequence with Deep Learning. Cell *176*, 535–548.e24. https://doi.org/10.1016/j.cell.2018.12.015.
24. Wang, H., Bienz, M., Yan, X.-X., and Xu, W. (2023). Structural basis of the interaction between BCL9-Pygo and LDB-SSBP complexes in assembling the Wnt enhanceosome. Nat. Commun. *14*, 3702. https://doi.org/10.1038/s41467-023-39439-9.
25. Bhati, M., Lee, C., Nancarrow, A.L., Lee, M., Craig, V.J., Bach, I., Guss, J.M., Mackay, J.P., and Matthews, J.M. (2008). Implementing the LIM code: the structural basis for cell type-specific assembly of LIM-homeodomain complexes. EMBO J. *27*, 2018–2029. https://doi.org/10.1038/emboj.2008.123.
26. Guex, N., and Peitsch, M.C. (1997). SWISS-MODEL and the Swiss-Pdb Viewer: An environment for comparative protein modeling. ELECTROPHORESIS *18*, 2714–2723. https://doi.org/10.1002/elps.1150181505.
27. Sayle, R., and Milner-White, E.J. (1995). RASMOL: biomolecular graphics for all. Trends Biochem. Sci. *20*, 374–376. https://doi.org/10.1016/S0968-0004(00)89080-5.
28. Brand, A.H., and Perrimon, N. (1993). Targeted gene expression as a means of altering cell fates and generating dominant phenotypes. Development *118*, 401–415. https://doi.org/10.1242/dev.118.2.401.
29. Ni, J.-Q., Liu, L.-P., Binari, R., Hardy, R., Shim, H.-S., Cavallaro, A., Booker, M., Pfeiffer, B.D., Markstein, M., Wang, H., et al. (2009). A Drosophila Resource of Transgenic RNAi Lines for Neurogenetics. Genetics *182*, 1089–1100. https://doi.org/10.1534/genetics.109.103630.

30. Palladino, M.J., Hadley, T.J., and Ganetzky, B. (2002). Temperature-Sensitive Paralytic Mutants Are Enriched For Those Causing Neurodegeneration in Drosophila. Genetics *161*, 1197–1208. https://doi.org/10.1093/genetics/161.3.1197.
31. Straub, J., Konrad, E.D.H., Grüner, J., Toutain, A., Bok, L.A., Cho, M.T., Crawford, H.P., Dubbs, H., Douglas, G., Jobling, R., et al. (2018). Missense Variants in RHOBTB2 Cause a Developmental and Epileptic Encephalopathy in Humans, and Altered Levels Cause Neurological Defects in Drosophila. Am. J. Hum. Genet. *102*, 44–57. https://doi.org/10.1016/j.ajhg.2017.11.008.
32. Hendricks, J.C., Finn, S.M., Panckeri, K.A., Chavkin, J., Williams, J.A., Sehgal, A., and Pack, A.I. (2000). Rest in *Drosophila* Is a Sleep-like State. Neuron *25*, 129–138. https://doi.org/10.1016/S0896-6273(00)80877-6.
33. Shaw, P.J., Cirelli, C., Greenspan, R.J., and Tononi, G. (2000). Correlates of Sleep and Waking in Drosophila melanogaster. Science *287*, 1834–1837. https://doi.org/10.1126/science.287.5459.1834.
34. Donelson, N., Kim, E.Z., Slawson, J.B., Vecsey, C.G., Huber, R., and Griffith, L.C. (2012). High-Resolution Positional Tracking for Long-Term Analysis of Drosophila Sleep and Locomotion Using the "Tracker" Program. PLoS One *7*, e37250. https://doi.org/10.1371/journal.pone.0037250.
35. Chen, S., Francioli, L.C., Goodrich, J.K., Collins, R.L., Kanai, M., Wang, Q., Alföldi, J., Watts, N.A., Vittal, C., Gauthier, L.D., et al. (2024). A genomic mutational constraint map using variation in 76,156 human genomes. Nature *625*, 92–100. https://doi.org/10.1038/s41586-023-06045-0.
36. Bruni, O., Breda, M., Mammarella, V., Mogavero, M.P., and Ferri, R. (2025). Sleep and circadian disturbances in children with neurodevelopmental disorders. Nat. Rev. Neurol. *21*, 103–120. https://doi.org/10.1038/s41582-024-01052-9.
37. von Frowein, J., Wizenmann, A., and Götz, M. (2006). The transcription factors Emx1 and Emx2 suppress choroid plexus development and promote neuroepithelial cell fate. Dev. Biol. *296*, 239–252. https://doi.org/10.1016/j.ydbio.2006.04.461.
38. Kinare, V., Iyer, A., Padmanabhan, H., Godbole, G., Khan, T., Khatri, Z., Maheshwari, U., Muralidharan, B., and Tole, S. (2020). An evolutionarily conserved Lhx2-Ldb1 interaction regulates the acquisition of hippocampal cell fate and regional identity. Development *147*, dev187856. https://doi.org/10.1242/dev.187856.
39. Howard, P.W., Jue, S.F., Ransom, D.G., and Maurer, R.A. (2010). Regulation of LIM-domain-binding 1 protein expression by ubiquitination of Lys134. Biochem. J. *429*, 127–136. https://doi.org/10.1042/BJ20091461.
40. Krivega, I., Dale, R.K., and Dean, A. (2014). Role of LDB1 in the transition from chromatin looping to transcription activation. Genes Dev. *28*, 1278–1290. https://doi.org/10.1101/gad.239749.114.
41. Mensah, M.A., Niskanen, H., Magalhaes, A.P., Basu, S., Kircher, M., Sczakiel, H.L., Reiter, A.M.V., Elsner, J., Meinecke, P., Biskup, S., et al. (2023). Aberrant phase separation and nucleolar dysfunction in rare genetic diseases. Nature *614*, 564–571. https://doi.org/10.1038/s41586-022-05682-1.
42. Bach, I., Carrière, C., Ostendorff, H.P., Andersen, B., and Rosenfeld, M.G. (1997). A family of LIM domain-associated cofactors confer transcriptional synergism between LIM and Otx homeodomain proteins. Genes Dev. *11*, 1370–1380. https://doi.org/10.1101/gad.11.11.1370.
43. Lee, S.-K., and Pfaff, S.L. (2003). Synchronization of Neurogenesis and Motor Neuron Specification by Direct Coupling of bHLH and Homeodomain Transcription Factors. Neuron *38*, 731–745. https://doi.org/10.1016/S0896-6273(03)00296-4.

The American Journal of Human Genetics, Volume *113*

Supplemental information

De novo variants in *LDB1* are linked to distinct neurodevelopmental phenotypes determined by variant location and differing pathomechanisms

Rebecca Fluri, Mireia Coll-Tané, Theresa Brunet, Benjamin Cogne, Solene Conrad, Mathilde Nizon, Francesco Nicita, Lorena Travaglini, Antonio Novelli, Margie Glissmeyer, Amanda Peterson, Jillian G. Buchan, Dan Serber, Kolja Meier, Jutta Gärtner, Susann Diegmann, Veronique Pingault, Tania Attie-Bitach, Thomas Courtin, Michael C. Schneider, Wing Hung, Inderneel Sahai, Lauren O'Grady, Katharina Steindl, Sarju G. Mehta, Christel Depienne, Delphine Heron, Boris Keren, Solveig Heide, Shane McKee, Franco Laccone, Lisa M. Dyer, Catherine Melver, Connie Motter, Wendy D. Jones, Zoey Trueblood Wilson, Divya Vats, Kristina Huß, Christiane Zweier, Heinrich Sticht, and Anne Gregor

Supplemental Information

Supplemental Figures

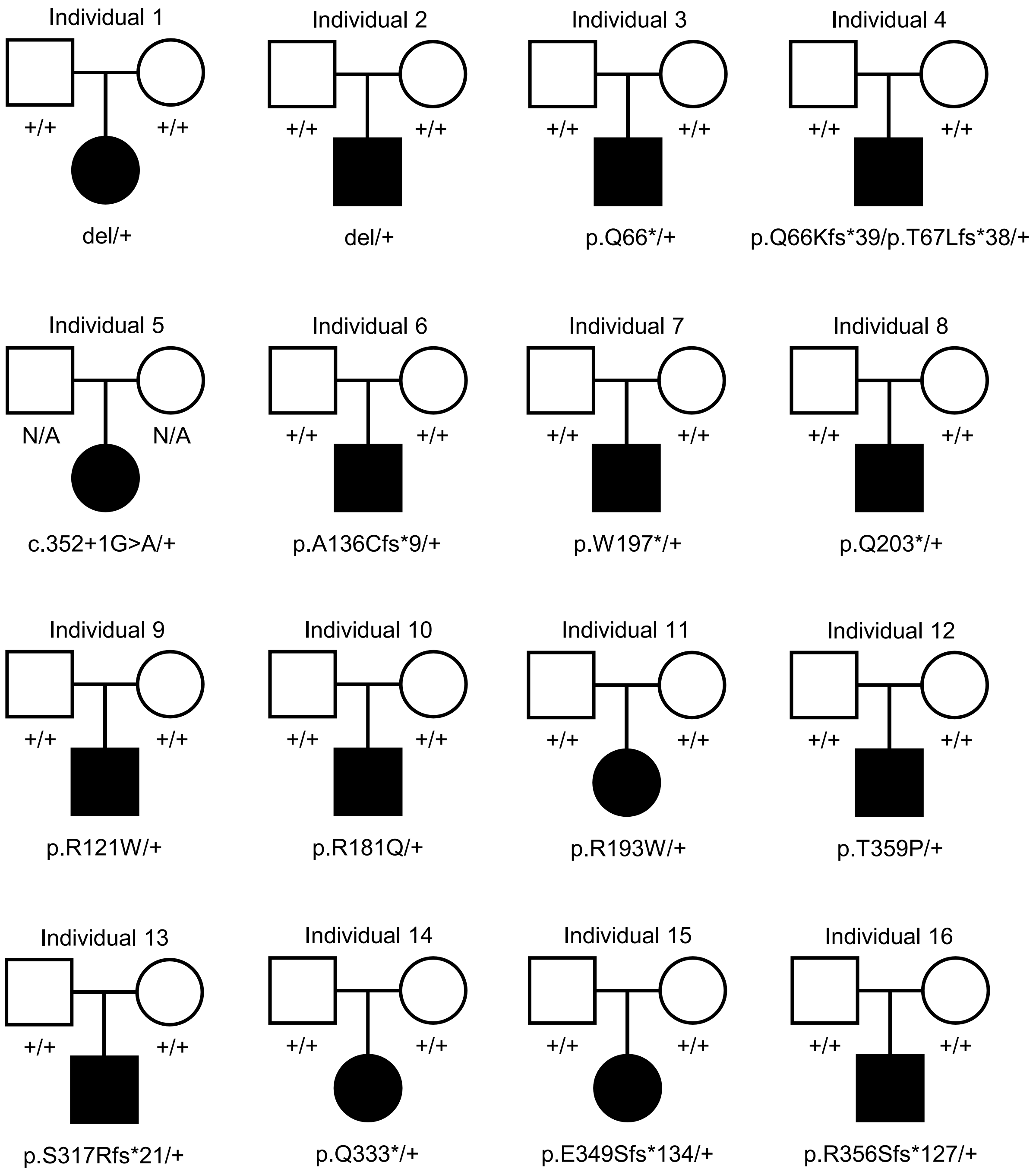

Figure S1. Pedigrees of all affected individuals included in this study.

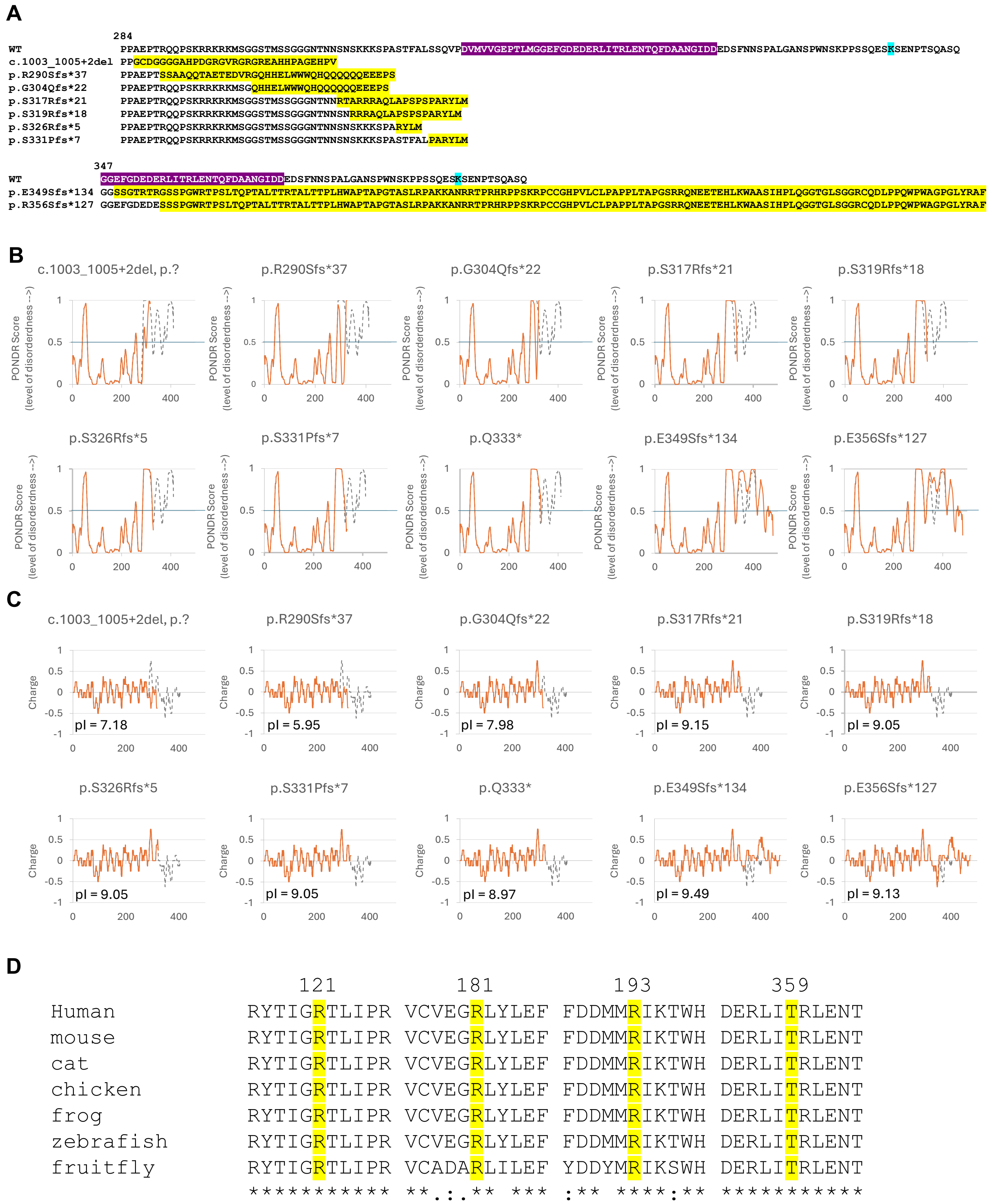

Figure S2. Changed C-terminal tail upon C-terminal LID-disrupting variants and conservation of *LDB1* missense variant positions.

(A) Published and novel C-terminal LID-disrupting frameshift variants alter C-terminal tails of LDB1 (alterations highlighted in yellow for each variant), omitting the ubiquitination site (marked in blue in the wild-type (WT) sequence) and at least part of the LID (highlighted in purple).

(B) Many C-terminal LID-disrupting variants reduce disorderdness of the C-terminus. Analysis was done using PONDR. Wild-type is shown as grey dashed line, respective mutants in orange.

(C) Charge analysis shows that most C-terminal LID-disrupting variants reduce acidity of LDB1 and increase the isoelectric point of the protein ((pI); pI wild-type LDB1 = 6.52). Analysis was done using the EMBOSS Charge tool for protein charge and the Expasy compute pI tool for isoelectric point. Wild-type is shown as grey dashed line, respective mutants in orange.

(D) All identified missense variants in LDB1 affect residues that are highly conserved. Multiple species alignment was done with Clustalw.[1] Reference sequences used were: human: NP_001106878.1, mouse: NP_001106879.1, cat: XP_023096621.1, chicken: XP_046775883.1, frog: NP_001090450.1, fruit fly: AAB62574.1. Affected amino acids are highlighted in yellow.

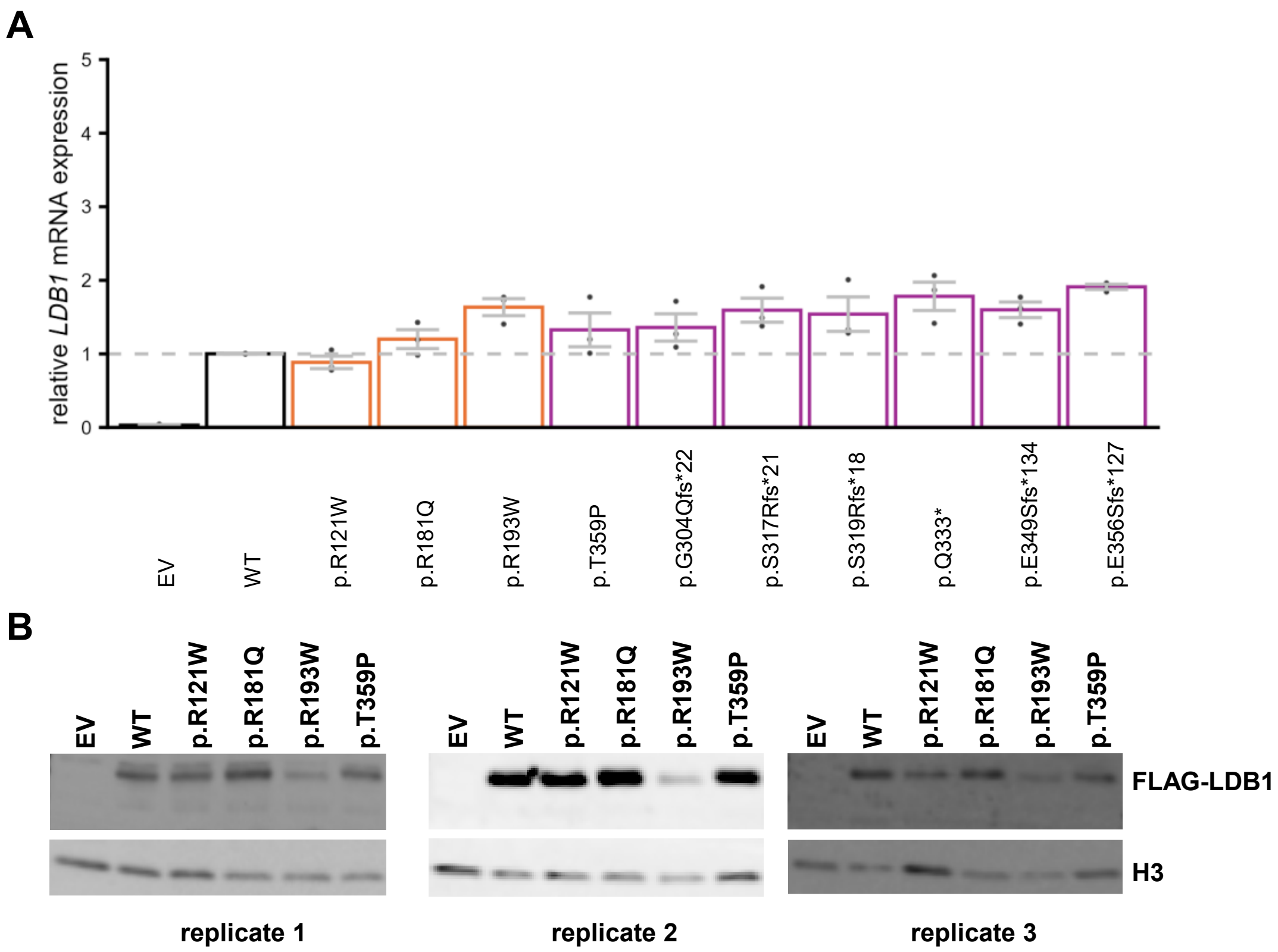

Figure S3. Expression level analysis of *LDB1* variants.

(A) Transcript levels of transfected wild-type (WT) or mutant *LDB1* were mostly comparable between different constructs and did not correlate with protein levels (see Figure 3B). QPCR analysis was carried out in three biological replicates with four technical replicates each; data were normalized to expression of WT *LDB1*; individual data points (biological replicates) are shown as black dots. EV – empty vector.

(B) Blots of three biological replicates of protein analysis for WT LDB1 and missense variants without MG132 treatment show consistent reduction of LDB1 for missense variant p.(Arg193Trp). Quantification and a representative image with the fourth replicate are shown in Figure 3B.

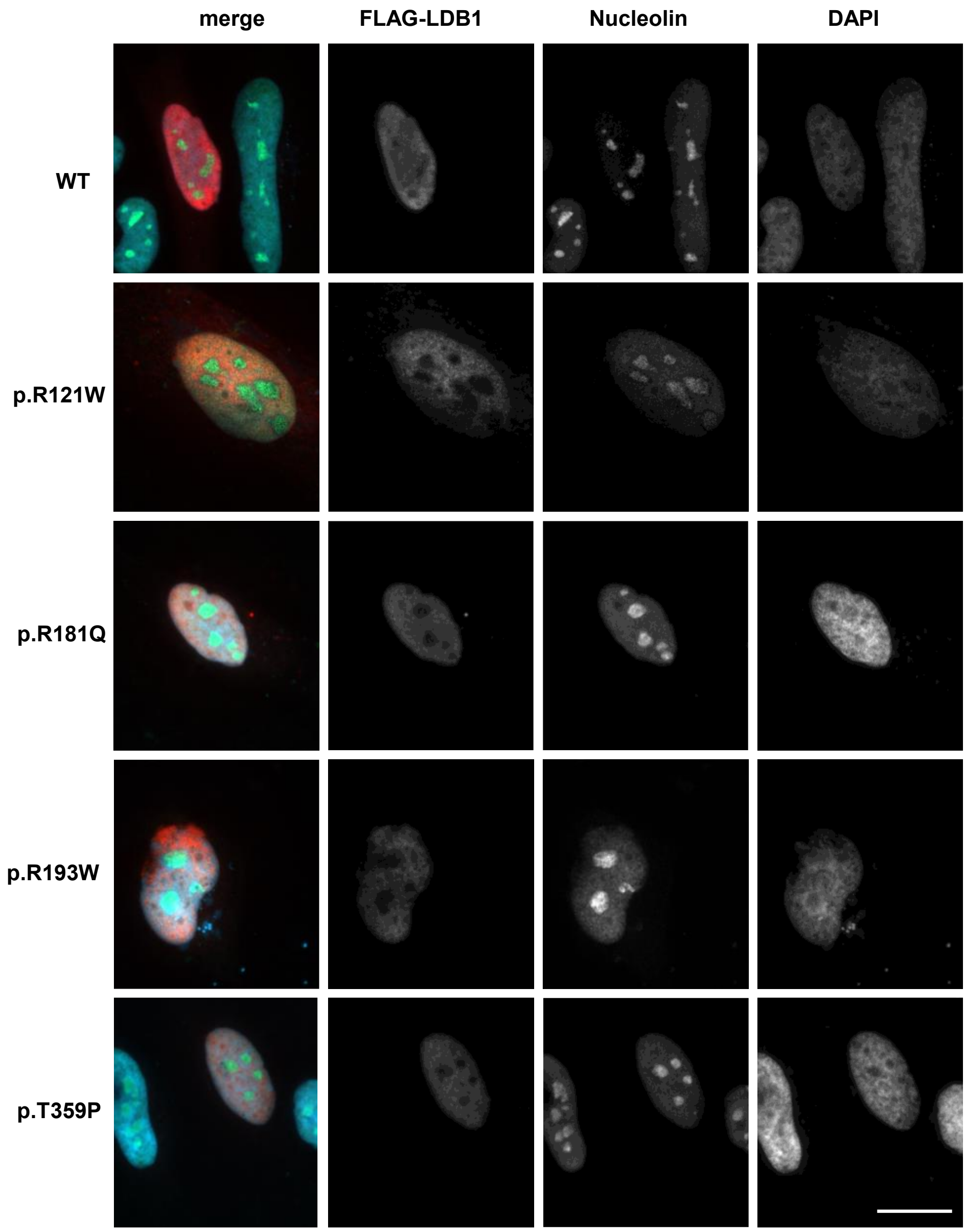

Figure S4. Immunofluorescence analysis of LDB1 missense variants.

Diffuse cytoplasmic localization of overexpressed FLAG-LDB1 in the nucleus of HeLa cells is not affected by LDB1 missense variants. Cells were stained with anti-FLAG (red) and anti-nucleolin (green) antibodies and nuclei were counterstained with DAPI. At least 50 cells were analyzed per condition. Images were taken on a Axioimager Z2 with a 63x objective, scale bar = 20μm.

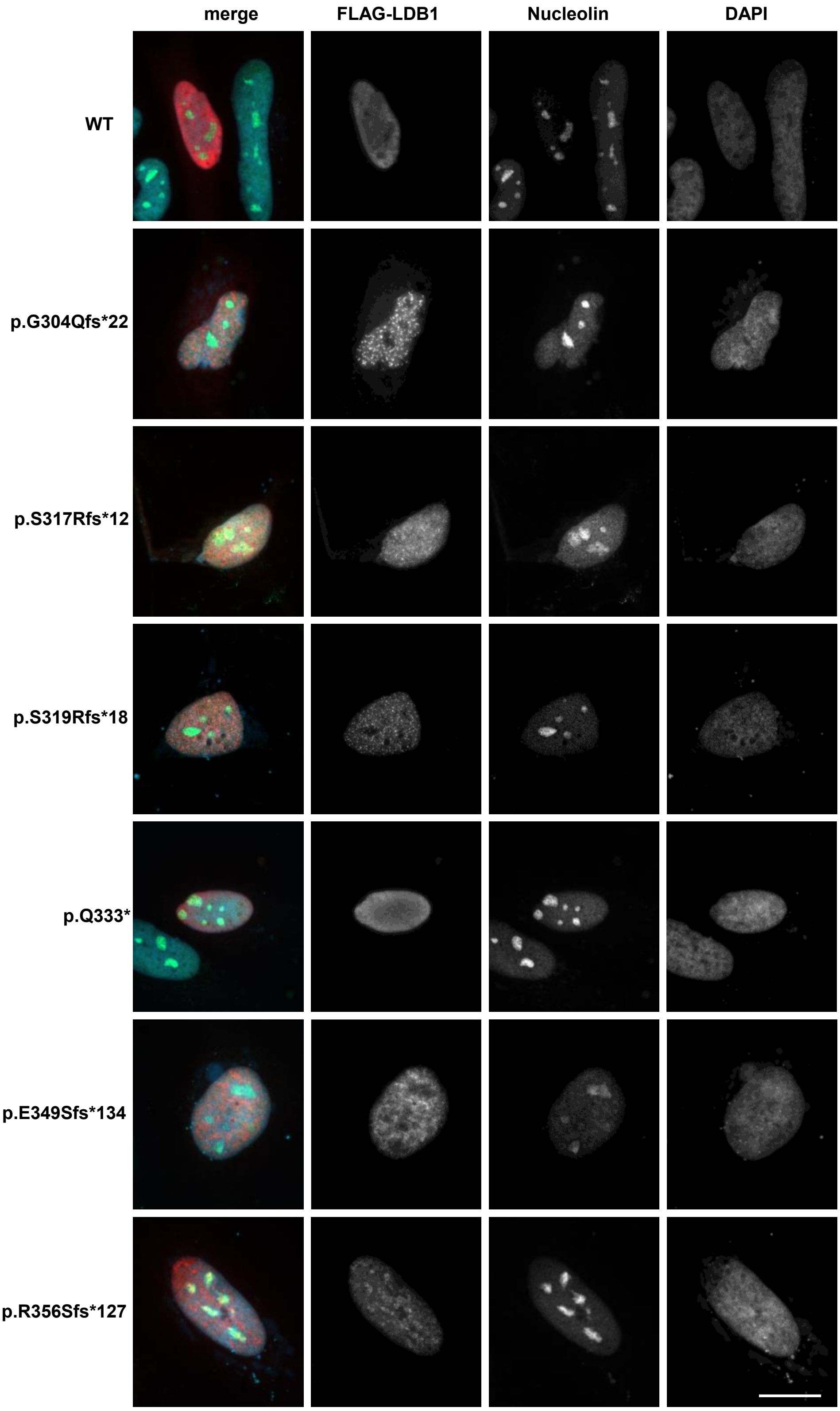

Figure S5. Immunofluorescence analysis of C-terminal LID-disrupting LDB1 variants.

All C-terminal frameshift variants show punctate nuclear aggregates of LDB1 that do not co-localize with the nucleolus. No aggregates are found for the nonsense variant p.(Gln333*). Cells were stained with anti-FLAG (red) and anti-nucleolin (green) antibodies and nuclei were counterstained with DAPI. At least 50 cells were analyzed per condition. Images were taken on a Axioimager Z2 with a 63x objective, scale bar = 20µm.

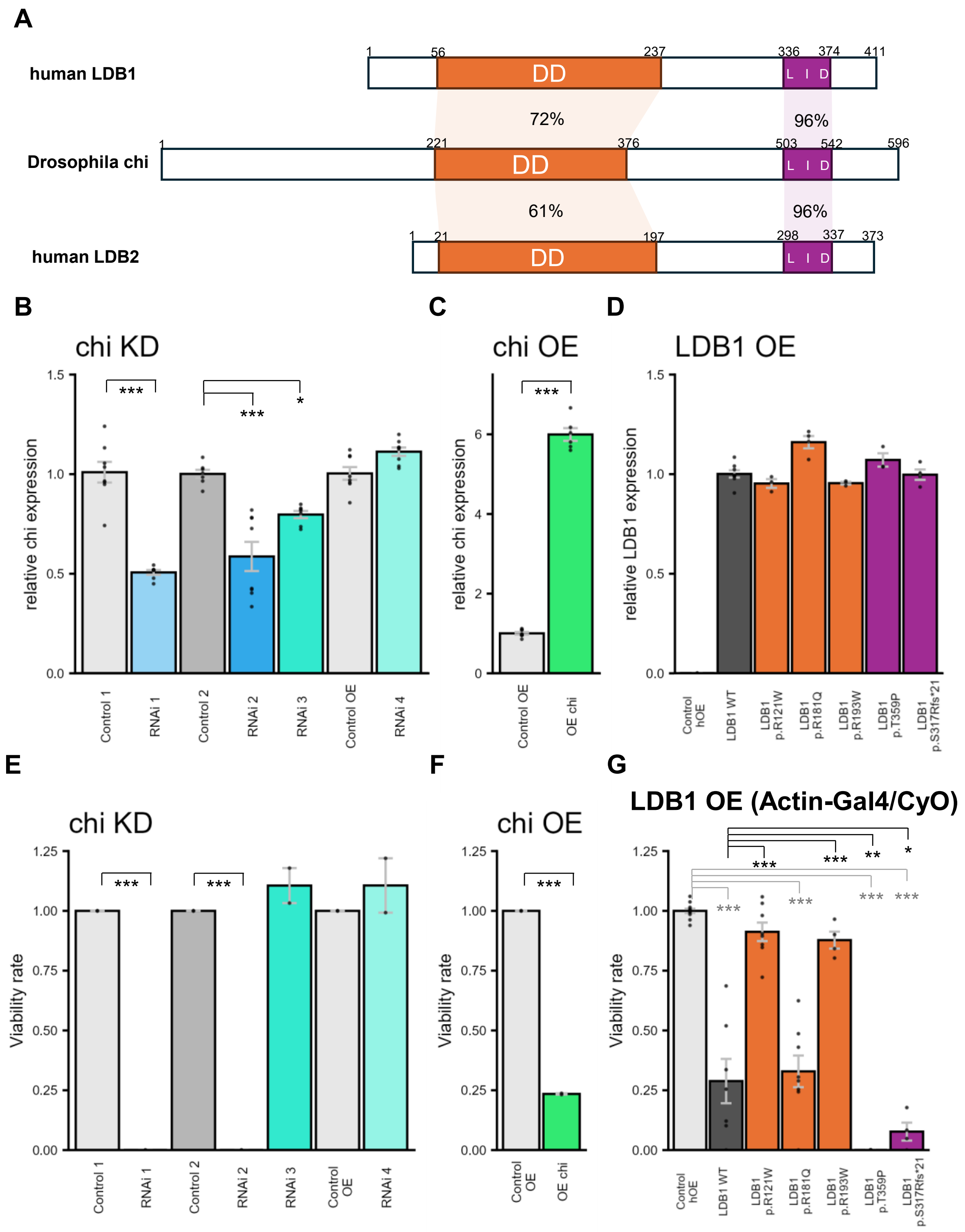

Figure S6. Expression and viability analysis of *chi/LDB1 Drosophila* lines.

(A) Schematic overview of human LDB1 and LDB2 as well as *Drosophila* chi, showing conservation of functional domains. DD – dimerization domain, LID – LIM interacting domain.

(B-D) Expression levels of *chi/LDB1* upon ubiquitous dosage manipulation were measured using qPCR. Knockdown of *chi* was confirmed for RNAi 1 and 2 (B) and overexpression of *chi* was confirmed for OE chi (C). Overexpression levels of human wildtype and mutant *LDB1* are

comparable for all lines (D). Analysis was carried out in at least 4 technical replicates; data were normalized to respective references: RNAi 1 to Control 1, RNAi 2 & 3 to Control 2, RNAi 4 & OE chi to Control OE and for overexpression of human LDB1 to LDB1 WT (see Table S3 for description of lines and matching controls); individual data points are shown as black dots and significance in B) & C) was calculated using a 1-sample student's t-test with a theoretical value of 1 (* $p < 0.05$, *** $p < 0.001$).

(E-F) Viability analysis of ubiquitous *chi* knockdown (E) or overexpression (F) with the actin-GAL4 / Tm3 Sb Tb driver showed complete lethality for RNAi lines 1 and 2 (E) and partial lethality for OE chi (F). Viability was not affected in RNAi 3 and 4, consistent with less or absent knockdown of *chi* (see (B)). Viability was normalized to that of respective Control lines set to 1. Significance was calculated using a student's t-test on the ratio of knockdown/overexpression vs. Balancer flies (*** $p < 0.001$).

(G) Confirmation of lethality phenotype of overexpression of human LDB1 with a second driver line in addition to data shown in Figure 4C. Viability of male flies upon ubiquitous overexpression (using actin-GAL4 /CyO driver) of human wild-type of mutant LDB1 was reduced for wild-type and p.(Arg181Gln), and even more strongly reduced for variants p.(Thr359Pro) and p.(Ser317Argfs*21). Viability was normalized to the Control line (Control hOE), which was set to 1. The experiment was carried out independently at least three times in duplicates each. At least 100 flies were counted per experiment and genotype. Significance was calculated on the ratios of non-CyO (overexpression) vs. CyO (Balancer) flies using a student's t-test with Bonferroni correction for multiple testing (* $p < 0.05$, ** $p < 0.01$, *** $p < 0.001$).

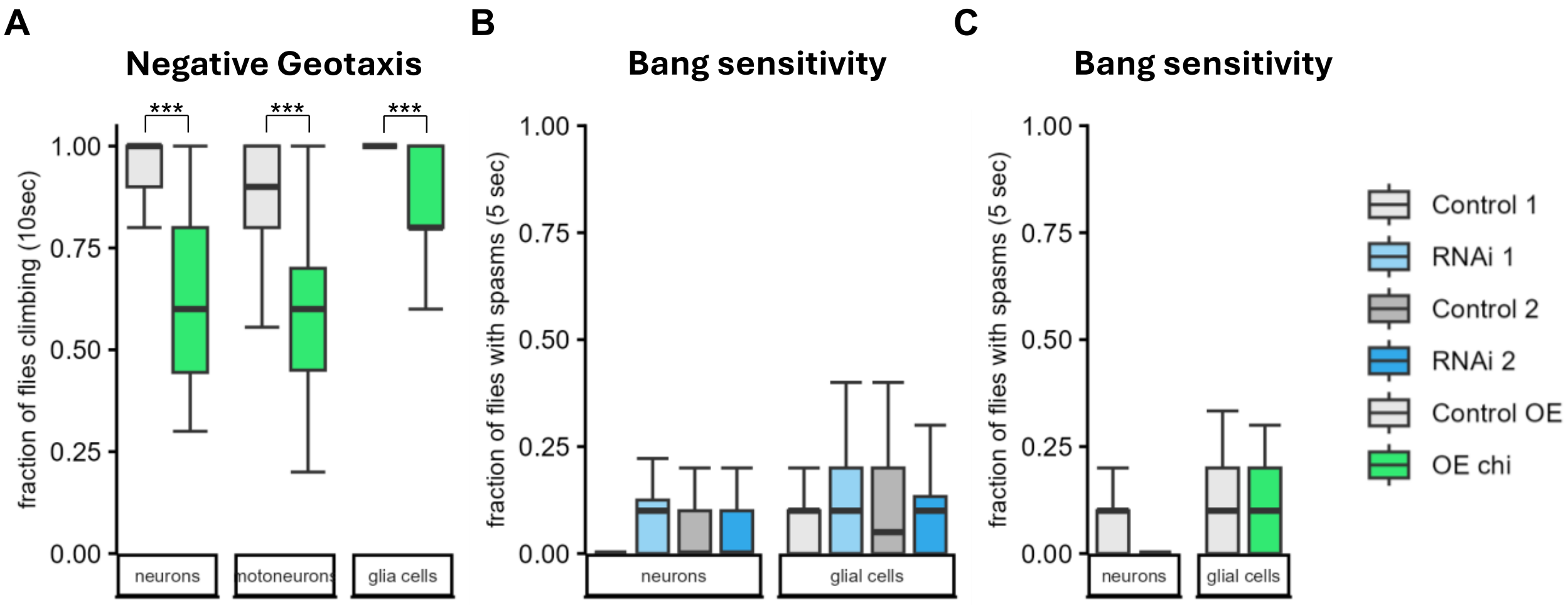

Figure S7. Negative geotaxis and bang sensitivity results of upon *chi* knockdown and overexpression.

(A) Climbing ability as measured with negative geotaxis assay was impaired upon pan-neuronal, motoneuron-specific and glial overexpression of *chi*. Fraction of flies able to climb 8 cm in 10sec after being tapped to the bottom of the vial was assessed.

(B-C) Seizure susceptibility is not increased upon pan-neuronal or glial knockown (B) or overexpression (C) of *chi* as measured with bang sensitivity assay. Fraction of flies showing spasms or paralysis 5 sec after 10sec of vortexing was measured.

At least 100 flies in batches of 10 (n= 10) were tested per experiment and condition. Significance was calculated using a Wilcoxon rank-sum test (*** $p < 0.001$).

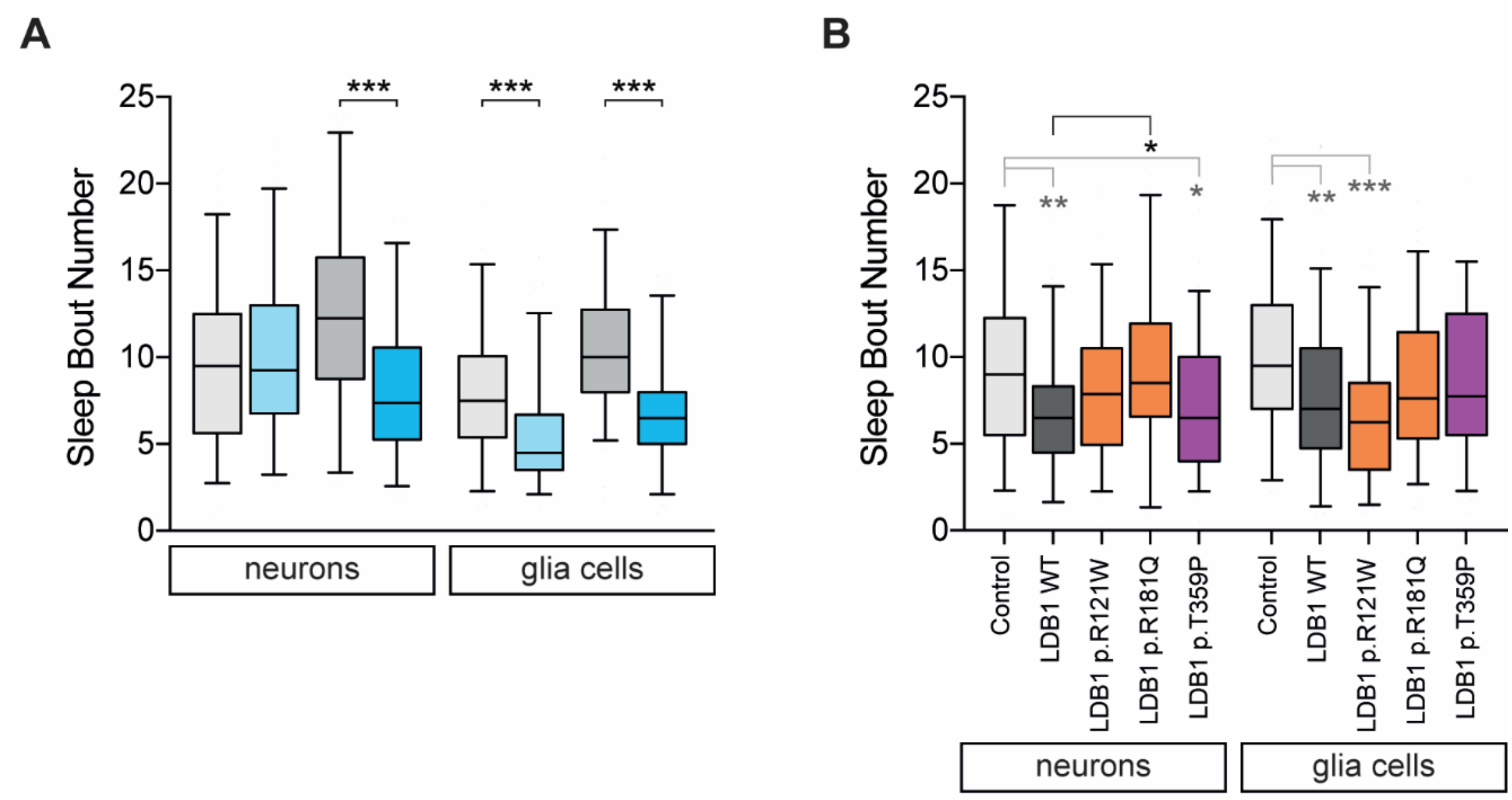

Figure S8. Sleep analysis shows alterations of sleep pattern upon *chi* knockdown and *LDB1* overexpression.

(A) Average number of sleep bouts during the dark period (ZT12-24). Pan-neuronal *chi* knockdown males with RNAi 2 exhibit fewer sleep bouts compared to isogenic controls. RNAi 1 shows no significant difference relative to controls. *Chi* knockdown in glial cells using both RNAi lines results in fewer sleep episodes compared to their respective background controls.

(B) Average number of sleep bouts during the dark period (ZT12–24). Neuronal overexpression of wild-type *LDB1* reduces sleep bouts compared to controls. Pan-neuronal overexpression of LDB1 carrying the R181Q variant significantly increases sleep bout frequency compared to wildtype LDB1 overexpression. Glial overexpression of wild-type LDB1 and the R121W variant reduces sleep bouts compared to controls. Data are represented as boxplots (25th-75th percentiles, median line; whiskers indicate 5th-95th percentiles). At least n = 40-70 flies were tested per genotype. Mann-Whitney or ANOVA tests, with Bonferroni correction for multiple comparisons. Significance: * $p < 0.05$, ** $p < 0.01$, *** $p < 0.001$.

Supplemental Tables

Table S1. Overview of detailed molecular and clinical information on affected individuals.

See excel file Table S1_S4.

Table S2. Overview of all antibodies used in this study

Target	Species	Catalog #	Supplier	Dilution
FLAG	rabbit	F7425	Sigma-Alrich	1:5’000 (WB)
FLAG	mouse	F1804	Sigma-Aldrich	1:500 (IF)
Myc	rabbit	2272	CST	1:2’500 (WB)
HA	rabbit	3724	CST	1:2’000 (WB)
Histone H3	rabbit	4499	CST	1:10’000 (WB)
Nucleolin	rabbit	14574	CST	1:100 (IF)
anti-rabbit-HRP	goat	170-6515	Bio-Rad	1:15’000
Alexa Fluor 488 anti-rabbit	goat	A11008	Thermo	1:500 (IF)
Alexa Fluor 546 anti-mouse	donkey	A10036	Thermo	1:500 (IF)

Table S3. Overview of all *Drosophila* lines used in this study

Fly Line	Description	Order #	Vendor
RNAi 1	knockdown of chi	107314	VDRC
RNAi 2	knockdown of chi	31049	BDSC
RNAi 3	knockdown of chi	35435	BDSC
RNAi 4	knockdown of chi	30454	VDRC
OE chi	overexpression of chi	67741	BDSC
Control 1	control for RNAi 1	60100	VDRC
Control 2	control for RNAi 2 and 3	36303	BDSC
Control OE	control for OE chi and RNAi 4	60000	VDRC
Control hOE	control for human OE lines	24749	BDSC
LDB1 WT	human OE	custom-made	FlyORF
LDB1 p.R121W	human OE	custom-made	FlyORF
LDB1 p.R181Q	human OE	custom-made	FlyORF
LDB1 p.R193W	human OE	custom-made	FlyORF
LDB1 p.T359P	human OE	custom-made	FlyORF
LDB1 p.S317Rfs*21	human OE	custom-made	FlyORF
RNAi 1 ; LDB1 WT	human OE and chi KD	custom-made	assembled in-house
RNAi 1 ; LDB1 p.R121W	human OE and chi KD	custom-made	assembled in-house
RNAi 1 ; LDB1 p.R181Q	human OE and chi KD	custom-made	assembled in-house
RNAi 1 ; LDB1 p.T359P	human OE and chi KD	custom-made	assembled in-house
Actin-GAL4/Tm3 Sb Tb	ubiquitous driver	3954	BDSC
Actin-GAL4/CyO	ubiquitous driver	4414	BDSC
Elav-GAL4/CyO	pan-neuronal driver, used for Geotaxis and Bang sensitivity	8765	BDSC
Elav-GAL4	pan-neuronal driver, used for sleep analysis	8760	BDSC
D42-GAL4	motoneuron driver	8816	BDSC
Repo-GAL4/Tm3 Sb	glial driver, used for Geotaxis and Bang sensitivity	7415	BDSC
Repo-GAL4/Tm6 Sb Tb	glial driver, used for sleep analysis	re-balanced from 7415	assembled in-house
Kr/Cyo ; D/Tm6c Sb Tb	double balancer line for generation of rescue constructs		assembled in-house

KD – knockdown, OE – overexpression, hOE – human gene overexpression, WT – wild-type

Table S4. Overview of detailed molecular data for all variants including applied ACMG criteria, prediction scores and summarized results of functional assays.

See excel file Table_S1_S4.

References

1. Madeira, F., Madhusoodanan, N., Lee, J., Eusebi, A., Niewielska, A., Tivey, A.R.N., Lopez, R., and Butcher, S. (2024). The EMBL-EBI Job Dispatcher sequence analysis tools framework in 2024. Nucleic Acids Res *52*, W521–W525. https://doi.org/10.1093/nar/gkae241.
